# Oncoprotein HBXIP enhances HOXB13 acetylation and co-activates HOXB13 to confer tamoxifen resistance in breast cancer

**DOI:** 10.1186/s13045-018-0577-5

**Published:** 2018-02-23

**Authors:** Bowen Liu, Tianjiao Wang, Huawei Wang, Lu Zhang, Feifei Xu, Runping Fang, Leilei Li, Xiaoli Cai, Yue Wu, Weiying Zhang, Lihong Ye

**Affiliations:** State Key Laboratory of Medicinal Chemical Biology, Department of Biochemistry, College of Life Sciences, Nankai University, Tianjin, 300071 People’s Republic of China

**Keywords:** Breast cancer, Tamoxifen resistance, HBXIP, HOXB13, Acetylation, Chaperone-mediated autophagy

## Abstract

**Background:**

Resistance to tamoxifen (TAM) frequently occurs in the treatment of estrogen receptor positive (ER+) breast cancer. Accumulating evidences indicate that transcription factor HOXB13 is of great significance in TAM resistance. However, the regulation of HOXB13 in TAM-resistant breast cancer remains largely unexplored. Here, we were interested in the potential effect of HBXIP, an oncoprotein involved in the acceleration of cancer progression, on the modulation of HOXB13 in TAM resistance of breast cancer.

**Methods:**

The Kaplan-Meier plotter cancer database and GEO dataset were used to analyze the association between HBXIP expression and relapse-free survival. The correlation of HBXIP and HOXB13 in ER+ breast cancer was assessed by human tissue microarray. Immunoblotting analysis, qRT-PCR assay, immunofluorescence staining, Co-IP assay, ChIP assay, luciferase reporter gene assay, cell viability assay, and colony formation assay were performed to explore the possible molecular mechanism by which HBXIP modulates HOXB13. Cell viability assay, xenograft assay, and immunohistochemistry staining analysis were utilized to evaluate the effect of the HBXIP/HOXB13 axis on the facilitation of TAM resistance in vitro and in vivo.

**Results:**

The analysis of the Kaplan-Meier plotter and the GEO dataset showed that mono-TAM-treated breast cancer patients with higher HBXIP expression levels had shorter relapse-free survivals than patients with lower HBXIP expression levels. Overexpression of HBXIP induced TAM resistance in ER+ breast cancer cells. The tissue microarray analysis revealed a positive association between the expression levels of HBXIP and HOXB13 in ER+ breast cancer patients. HBXIP elevated HOXB13 protein level in breast cancer cells. Mechanistically, HBXIP prevented chaperone-mediated autophagy (CMA)-dependent degradation of HOXB13 via enhancement of HOXB13 acetylation at the lysine 277 residue, causing the accumulation of HOXB13. Moreover, HBXIP was able to act as a co-activator of HOXB13 to stimulate interleukin (IL)-6 transcription in the promotion of TAM resistance. Interestingly, aspirin (ASA) suppressed the HBXIP/HOXB13 axis by decreasing HBXIP expression, overcoming TAM resistance in vitro and in vivo.

**Conclusions:**

Our study highlights that HBXIP enhances HOXB13 acetylation to prevent HOXB13 degradation and co-activates HOXB13 in the promotion of TAM resistance of breast cancer. Therapeutically, ASA can serve as a potential candidate for reversing TAM resistance by inhibiting HBXIP expression.

**Electronic supplementary material:**

The online version of this article (10.1186/s13045-018-0577-5) contains supplementary material, which is available to authorized users.

## Background

Breast cancer is the most common malignant tumor, with high incidence and mortality rates in females worldwide [1]. It has been reported that approximately three-quarters of breast cancer patients express estrogen receptor alpha (ER-α) [1, 2]. Tamoxifen (TAM), a classic anti-estrogen drug, is able to antagonize estrogen by competitively binding to ER-α and assisting in the recruitment of co-repressors, leading to the suppression of ER-α-responsive genes [3–5]. However, the frequent occurrence of TAM resistance in patients has become a major obstacle for breast cancer therapy [6, 7]. An appropriate biomarker is important and useful for predicting response to TAM therapy. It has been reported that HOXB13 collaborates with activated ras to promote cell survival and proliferation in ovarian cancer [8]. Kim et al. reveal that HOXB13 acts as a modulator of intracellular zinc levels to facilitate prostate cancer metastasis [9]. In ER+ breast cancer, a high expression level of HOXB13 is associated with a more aggressive clinical course [10]. Extensive studies have elucidated that the expression ratio of HOXB13:IL17BR has been identified to predict the outcome of TAM monotherapy [10–13]. A report has disclosed that HOXB13 alone can predict the response to TAM therapy [14]. Jerevall et al. have revealed that increased HOXB13 protein is correlated with decreased benefit from TAM [15]. Studies indicate that HOXB13 expression level may be useful for identifying appropriate therapeutic regimens in breast cancer [10, 11, 15]. Much is known about the significance of HOXB13 in the prediction of TAM resistance, but the underlying mechanism of HOXB13 expression regulation in TAM resistance remains unexplored.

Mammalian hepatitis B X-interacting protein (HBXIP), also known as LAMTOR5 [16], is a conserved protein among mammalian species and is frequently expressed in multiple tissues [17, 18]. It has been reported that HBXIP is highly expressed in breast cancer tissues and functions as an oncoprotein in the development of breast cancer, such as proliferation, migration, and lipid metabolism reprogramming [19–21]. Bar-Peled’s group has discovered that HBXIP functions as a regulatory component in the amino acid-induced activation of mTORC1 [16]. Recently, HBXIP has been proven to regulate the expression of cancer-associated genes by acting as a co-activator of different transcription factors [22–24]. However, whether HBXIP can affect the response to TAM therapy in breast cancer remains unknown.

In search of new solutions to cancer, it is common to use well-known drugs to treat other diseases that may improve therapeutic outcome. Khuder’s group reported that ER+ breast cancer patients who regularly consume nonsteroidal anti-inflammatory drugs (NSAIDs) present a 22% decline in breast cancer risk [25]. Aspirin (ASA), as a classic NSAID, has been widely revealed to play novel roles in the reduction of incidences of cancers such as colorectal cancer, liver cancer, and breast cancer [26–28]. It has been revealed that HBXIP can serve as an upstream controller to activate some downstream effectors of ASA treatment including cyclooxygenase 2 (COX2), NF-kB, or extracellular signal-related kinase (ERK) [19, 29–31]. Here, we are wondering whether ASA is involved in the regulation of HBXIP in TAM resistance.

In this study, our finding shows that oncoprotein HBXIP can suppress chaperone-mediated autophagy (CMA)-dependent degradation of HOXB13 via enhancing its acetylation, resulting in the accumulation of HOXB13. Then, HBXIP acts as a co-activator of HOXB13 to stimulate IL-6 transcription in the facilitation of TAM resistance. Interestingly, we find that ASA can depress the HBXIP/HOXB13 axis via decreasing HBXIP expression, ameliorating TAM resistance.

## Methods

### Generation of stable cell lines and cell culture

The characteristics of each stable cell line are as follows: MCF-7-pCMV (stably transfected with pCMV-Tag2B empty vector), MCF-7-HBXIP (stably transfected with pCMV-HBXIP plasmid), BT474-pSilencer-Random (stably transfected with pSilencer 4.1 CMV vector containing a random fragment), and BT474-pSilencer-HBXIP (stably transfected with pSilencer 4.1 CMV vector containing the HBXIP RNAi fragment [17]). The cells were selected in the presence of 800 μg/ml G418 (Invitrogen) or 1 μg/ml puromycin (Invitrogen) and cultured with 400 μg/ml G418 or 0.5 μg/ml puromycin, respectively. MCF-7, T47D, BT474, and the above stable cell lines were maintained in RPMI Medium 1640 (Gibco, USA) with 10% FBS (Gibco). The human embryonic kidney cell lines 293T (HEK293T) and the breast cancer cell line MDA-MB-468 were maintained in DMEM (Gibco) supplemented with 10% FBS, 100 U/ml streptomycin, 1% glutamine, and 100 U/ml penicillin. The MCF-7, T47D, BT474, HEK293T, and MDA-MB-468 cell lines were obtained from the American Type Culture Collection (ATCC). Lipofectamine 2000 reagent (Invitrogen, USA) was used for the transfection experiments.

### Reagents and antibodies

Antibodies of this study were as follows: anti-HBXIP (Abcam, UK), anti-HOXB13 (Abcam), anti-β-actin (Sigma-Aldrich, USA), anti-Flag-tag (Sigma-Aldrich), anti-STAT3 (ImmunoWay Biotechnology Company, USA), anti-GFP (Sigma-Aldrich), anti-GCN5 (Proteintech, USA), anti-acetylated lysine (Aviva Systems Biology, CA), anti-p300 (Santa Cruz Biotechnology, USA), anti-ER-α (ImmunoWay Biotechnology Company), anti-HSC70 (Proteintech), anti-Ki67 (Santa Cruz Biotechnology), and IL-6 neutralizing antibody (Abcam). Trichostatin A (TSA) and cycloheximide (CHX) were separately purchased from Beyotime Biotechnology (China) and MedChem Express (USA). 4OH-TAM and ASA were purchased from Sigma-Aldrich.

### Plasmid construction and small interference RNA (siRNA)

pCMV-tag2B, pcDNA3.1 (+), pGL3-Basic, pRL-TK, pcDNA3.1(+)-HBXIP, and pCMV-tag2B-HBXIP were kept in our laboratory. The 5′ flanking region of the IL-6 gene was amplified by PCR from the genomic DNA of MCF-7 cells and was inserted into the KpnI/HindIII site in the pGL3-Basic vector to generate the pGL3-IL-6 construct. The complete human HOXB13 (NCBI reference sequence: NM_006361.5) cDNA sequence was subcloned into the pEGFP-C2 vector or the pCMV-tag2B vector to generate GFP-HOXB13 or pCMV-HOXB13. All siRNAs, the miR-520b inhibitor, and the control inhibitor were purchased from RiboBio Co., Ltd. (China). All siRNA sequences and related primers are listed in Additional file 1: Table S1.

### Immunohistochemistry staining analysis

The immunohistochemistry assay was performed as previously described [29]. For Ki67 staining, the slides were incubated with rabbit anti-Ki67 antibody (Santa Cruz Biotechnology) at 4 °C for overnight and treated with biotinylated secondary antibody at room temperature for 30 min. Immunostaining was developed by using chromogen 3,3′-diaminobenzidine (DAB) and counterstained with Mayer’s hematoxylin. The positive Ki67 staining was identified by Image-Pro Plus software. The breast cancer tissue microarray (No. AM08C22) containing samples from 57 primary breast carcinomas, 15 lymph node metastatic breast carcinomas, 6 normal breast tissues, and 21 breast diseases was purchased from Xi’an Aomei Biotechnology (China). For the antigen retrieval of HBXIP, the slides were boiled in 10 mM citrate buffer (pH 6.0) for 5 min. For HOXB13, the slides were placed in 0.05% trypsin antigen retrieval buffer with 0.05 M CaCl_2_ (pH 7.8) at 37 °C for 30 min. The staining levels of HBXIP and HOXB13 were classified into four groups using a modified scoring method based on the intensity of staining (0 = negative; 1 = low; 2 = moderate; 3 = high) and the percentage of stained cells (0 = 0% stained; 1 = 1–29% stained; 2 = 30–65% stained; 3 = 66–100% stained). A multiplied score (intensity score × percentage score) lower than 1 was considered to be negative staining (0); 1, 2, and 3 were considered to be weak staining (1); 4 and 6 were considered to be moderate staining (2); and 9 was considered to be intense staining (3). The patient records are presented in Additional file 2: Table S2.

### Patient samples

Thirty-four clinical ER+ breast cancer and noncancerous tissues were surgically resected and collected from the patients in Tianjin Tumor Hospital (Tianjin, China). Written informed consent was provided by the patients, approving the usage of their tissues for research. The study was approved by the Research Ethics Committee of Nankai University. The patient records are presented in Additional file 3: Table S3.

### Co-immunoprecipitation assay

The cells transfected with the corresponding plasmids were harvested and lysed in a lysis buffer (50 mM Tris-HCl pH 7.5, 150 mM NaCl, 1 mM EDTA, 0.5% Triton X-100, 10% glycerine, 1 mM protease inhibitor PMSF). The lysates were incubated with the anti-Flag M2 affinity gel (Sigma-Aldrich) or the anti-GFP affinity gel (MBL, Japan) at 4 °C for 4 h. After eight times of washing with the lysis buffer, the precipitated proteins were eluted from the gel by 0.1 M glycine-HCl (pH 3.0) buffer and neutralized with 1 M Tris-HCl (pH 7.5) containing 1.5 M NaCl then resolved by SDS-PAGE followed by immunoblotting. To detect the interaction of the endogenous proteins in BT474 cells, the cell lysate was incubated with rabbit anti-HBXIP, rabbit anti-HOXB13, or the negative control IgG along with protein G-Sepharose (Santa Cruz Biotechnology). The subsequent procedures were same as above.

### Xenograft

**Fig. 1:**
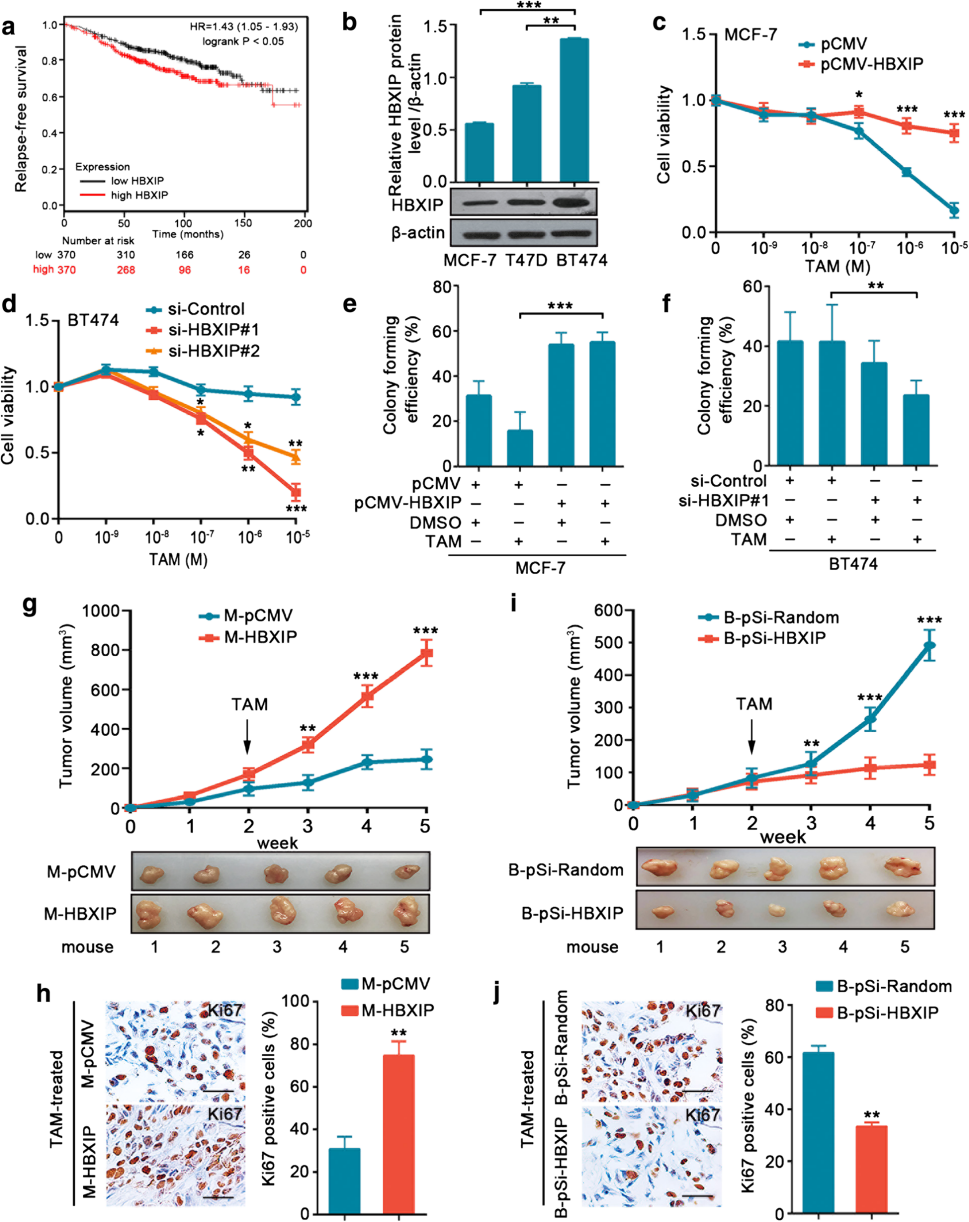
HBXIP contributes to TAM resistance in breast cancer. **a** Relapse-free survival analysis of TAM-treated ER+ breast cancer patients by the Kaplan-Meier plotter online resource (http://kmplot.com/analysis/). The plot was generated according to the HBXIP expression level (log-rank *P* = 0.021). **b** Immunoblotting analysis of HBXIP in MCF-7, T47D, and BT474 cells (lower panel) and the quantification of the intensity relative to β-actin (upper panel). **c**, **d** Cell viability assay in MCF-7 (**c**) and BT474 (**d**) cells treated with corresponding doses of TAM after being transiently transfected with the indicated plasmids or siRNAs. **e**, **f** Colony forming efficiencies of MCF-7 (**e**) and BT474 (**f**) cells treated with DMSO or TAM (1 μM) after being transiently transfected with the indicated plasmids or siRNA. **g**, **h** Growth curve and imaging (**g**), Ki67 (a cell proliferation marker) staining by IHC assay and the statistics of Ki67 positive cells (**h**) of the xenograft tumors derived from MCF-7-pCMV (called M-pCMV) or MCF-7-HBXIP (called M-HBXIP) cells (each group, *n* = 5). Scale bar, 50 μm. **i**, **j** Growth curve and imaging (**i**), Ki67 staining by IHC assay and the statistics of Ki67 positive cells (**j**) of the xenograft tumors derived from BT474-pSilencer-Random (called B-pSi-Random) or BT474-pSilencer-HBXIP (called B-pSi-HBXIP) cells (each group, *n* = 5). Scale bar, 50 μm. All experiments were repeated at least three times. Error bars represent ± SD. *P < 0.05, **P < 0.01, and ***P < 0.001 by two-tailed Student’s *t* test

Under the guideline of National Institutes of Health Guide for the Care and Use of Laboratory Animals, five- to six-week-old female BALB/c athymic nude mice were fed and housed. β-estradiol (Sigma) dissolved in olive oil (5 mg/ml, 0.1 ml) was administrated by gavage in − 3 day. On day 0, the cells were collected and suspended at the concentration of 5 × 10^6^ cells/ml in 0.2 ml of 1:1 PBS/Matrigel (BD Biosciences) and then injected into the fourth mammary fat pad (mfp) of each mouse. β-Estradiol supplementation was carried out twice a week. As seen in Fig. 1g, i, after two weeks, the mice were given TAM citrate (Yangzijiang company, China) (suspended in physiological saline, 5 mg/kg) by gavage daily. As shown in Fig. 7, the mice were randomized into four treatment groups when the tumor size reached about 150 mm^3^ and were treated daily by gavage with the indicated drug. Tumor volume and body weight were monitored twice a week. Post-treatment, the mice were euthanized, and necropsies were performed. Blind measurements were carried out to avoid unconscious biases. Tumor volume (V) was estimated by the formula: V = (length × width^2^) × 0.5.

### Statistical analysis

The statistical significance of in vitro and in vivo data was assessed by comparing mean values (± SD) using a two-tailed Student’s *t* test. The significance was set as *P < 0.05, **P < 0.01, and ***P < 0.001. In the GEO data analysis, the low and high groups of HBXIP expression were defined by comparing with the median of 90 mono-TAM-treated patients in GSE1456. The relapse-free survival in GSE1456 was analyzed by Gehan-Breslow-Wilcoxon test. The correlation between HBXIP and IL-6 mRNA levels in 34 ER+ clinical breast cancer tissues was determined by Pearson’s correlation coefficient. The association between HBXIP and HOXB13 expression in breast tissue microarray was statistically analyzed by Pearson chi-square independence test. Correlations and relapse-free survival were analyzed by Microsoft Access, SPSS 22.0, and Graph Pad Prism 6.0.

## Results

### HBXIP contributes to TAM resistance in breast cancer

The high incidence of tamoxifen (TAM) resistance is an impediment to the hormone therapy of breast cancer that should not be ignored [5]. To explore whether HBXIP was involved in TAM resistance, we assessed the correlation between HBXIP expression and the relapse-free survival of ER+ breast cancer patients with TAM treatment, by using the Kaplan-Meier plotter online resource [32]. The data showed that higher expression levels of HBXIP were associated with shorter relapse-free survival times (Fig. 1a). Meanwhile, the analysis of the GEO dataset (GSE1456) [33] in which breast cancer patients were treated with TAM monotherapy (*n* = 90) also displayed the inverse association of HBXIP expression and relapse-free survival (Additional file 4: Figure S1a). We confirmed the relationship of HBXIP and relapse-free survival in ER+ breast cancer by GOBO online resource [34] (Additional file 4: Figure S1b). The immunoblotting analysis demonstrated that HBXIP was more strongly expressed in the native TAM-resistant cell line BT474 than two TAM-sensitive cell lines, MCF-7 and T47D (Fig. 1b). Notably, overexpression of HBXIP induced TAM resistance in TAM-sensitive cells (Fig. 1c; Additional file 4: Figure S1c), while silencing HBXIP made TAM-resistant cells sensitive to TAM (Fig. 1d; Additional file 4: Figure S1d). Moreover, the elevation of the level of HBXIP in TAM-sensitive cells blocked the inhibition of colony formation mediated by TAM (Fig. 1e; Additional file 2: Figure S1e, g, h). However, the reduction of HBXIP in TAM-resistant cells made TAM effective again (Fig. 1f; Additional file 2: Figure S1f). To further validate the effect of HBXIP on TAM resistance in vivo, we constructed different stable breast cancer cell lines including MCF-7-pCMV, MCF-7-HBXIP, BT474-pSilencer-Random, and BT474-pSilencer-HBXIP (Additional file 2: Figure S1i). Strikingly, MCF-7-HBXIP xenografts grew rapidly in the mammary fat pad (mfp) of mice despite the presence of TAM (Fig. 1g, h; Additional file 2: Figure S1j). In contrast, compared with those of the BT474-pSilencer-Random group, the mfp xenografts of BT474-pSilencer-HBXIP group restored sensitivity to TAM (Fig. 1i, j; Additional file 2: Figure S1k). Thus, these data support that HBXIP contributes to TAM resistance in breast cancer.

### HBXIP induces TAM resistance by increasing the protein level of HOXB13

**Fig. 2:**
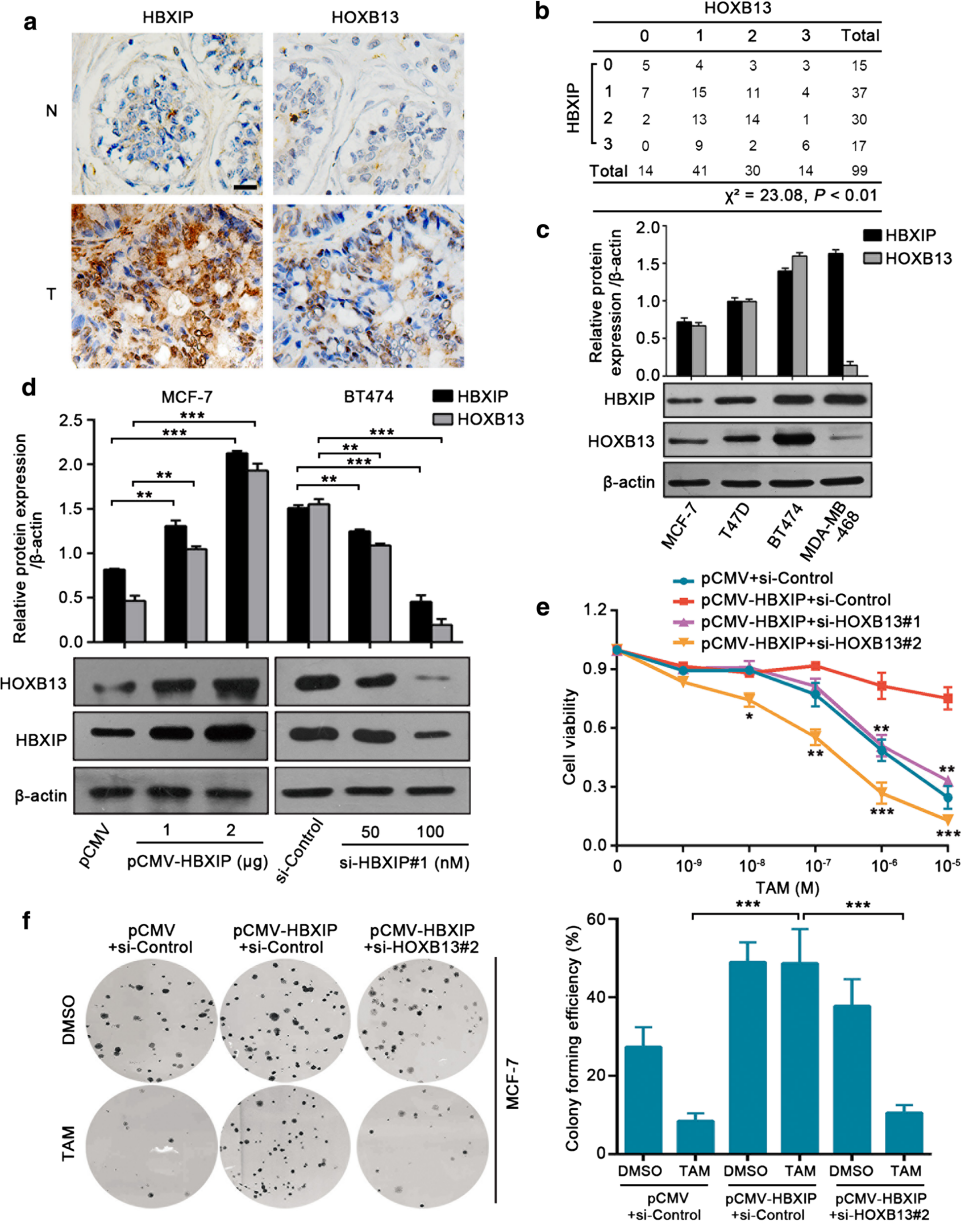
HBXIP induces TAM resistance by increasing the protein level of HOXB13. **a** IHC staining of HBXIP and HOXB13 in normal breast tissues (N) and breast carcinomas (T) from ER+ breast tissue microarray. Scale bar, 20 μm. **b** The association between HBXIP and HOXB13 expression levels in the abovementioned tissue microarray was statistically analyzed by Pearson chi-square independence test, *χ*^2^ = 23.08, *P* < 0.01. **c** Immunoblotting analysis of HBXIP and HOXB13 in different breast cancer cell lines (lower panel). The upper panel is the quantification of the intensity relative to β-actin. MDA-MB-468 is a triple-negative breast cancer cell line. **d** Immunoblotting analysis of HOXB13 in MCF-7 and BT474 cells transiently transfected with the indicated plasmids or siRNA (lower panel). The upper panel is the quantification of the intensity relative to β-actin. **e** Cell viability assay in MCF-7 cells treated with indicated concentrations of TAM after being transiently transfected with the displayed plasmids or siRNAs. Error bars represent ± SD. *P < 0.05, **P < 0.01, and ***P < 0.001 (HBXIP compared with HBXIP+si-HOXB13) by two-tailed Student’s *t* test. **f** A colony photograph and the colony forming efficiency of MCF-7 cells treated with DMSO or TAM (1 μM) after being transiently transfected with the displayed plasmids or siRNA. All experiments were repeated at least three times. Error bars represent ± SD. *P < 0.05, **P < 0.01, and ***P < 0.001 by two-tailed Student’s *t* test

Next, we explored the underlying mechanism by which HBXIP mediated TAM resistance. Jerevall’s group has reported that a high level of HOXB13 protein is associated with a decreased benefit from TAM therapy [15]. Thus, we considered whether HBXIP could modulate HOXB13, thereby facilitating TAM resistance. A tissue microarray containing 99 clinical ER+ breast tissues was used for HOXB13 and HBXIP staining, and the data indicated that HOXB13 was positively related to HBXIP in the examined specimens (Pearson chi-square independence test, *χ*^2^ = 23.08, *P* < 0.01. Fig. 2a, b; Additional file 1: Figure S2a; Additional file 2: Table S2). The immunoblotting analysis revealed a positive relationship between HBXIP and HOXB13 in three ER+ breast cancer cell lines, namely MCF-7, T47D, and BT474 (Fig. 2c). The qRT-PCR assay demonstrated that the overexpression and silencing HBXIP did not affect the mRNA level of HOXB13 (Additional file 2: Figure S2b, c). However, HBXIP markedly upregulated the protein level of HOXB13 (Fig. 2d). To explore the role of HOXB13 in HBXIP-induced TAM resistance, we performed cell viability and colony formation assays in MCF-7 and T47D cells with HBXIP overexpression and/or HOXB13 knockdown (Fig. 2e, f; Additional file 4: Figure S2d–f). We found that silencing HOXB13 significantly disrupted HBXIP-provoked cell proliferation and TAM resistance (Fig. 2e, f; Additional file 4: Figure S2e, f). Therefore, we conclude that HBXIP can induce TAM resistance by elevating the protein level of HOXB13 in breast cancer.

### HBXIP enhances acetylation of HOXB13 at K277 site via acetylase p300

**Fig. 3:**
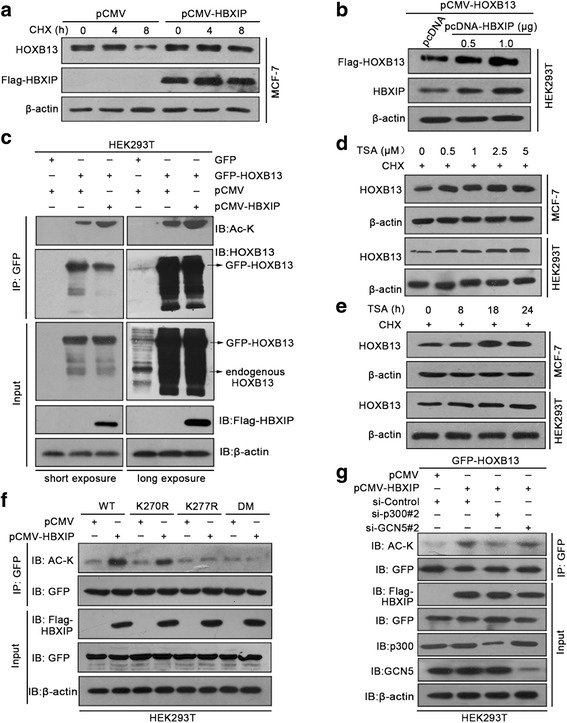
HBXIP enhances acetylation of HOXB13 at K277 site via acetylase p300. **a** Immunoblotting analysis of HOXB13 in MCF-7 cells time-dependently treated with 100 μg/ml cycloheximide (CHX) after being transiently transfected with the indicated plasmids. **b** Immunoblotting analysis of exogenous Flag-HOXB13 in HEK293T cells. The cells were transiently transfected with pCMV-HOXB13 accompanied by pcDNA or pcDNA-HBXIP. The protein level of Flag-HOXB13 was examined by the anti-Flag antibody. **c** Acetylation level of HOXB13 was detected by a Co-IP assay using GFP-beads and an immunoblotting analysis performed with the anti-acetyl-lysine antibody in HEK293T cells. The cells were transiently transfected with the indicated plasmids. The protein levels of HBXIP and HOXB13 were examined by anti-Flag or anti-HOXB13 antibodies, respectively. The left and right panels are identical results that differ in exposure time. **d**, **e** Immunoblotting analysis of HOXB13 in MCF-7 cells and HEK293T cells treated with 100 μg/ml CHX along with different concentrations of trichostatin A (TSA) for 18 h (**d**) or 1 μM TSA (**e**) for the indicated time points. **f** Acetylation level of HOXB13 was detected by a Co-IP assay using GFP-beads and an immunoblotting analysis performed with the anti-acetyl-lysine antibody in HEK293T cells. The cells were separately transiently transfected with GFP-HOXB13-WT, GFP-HOXB13-K270R, GFP-HOXB13-K277R, or GFP-HOXB13-DM along with pCMV or pCMV-HBXIP. The protein levels of HBXIP and HOXB13 were examined by anti-Flag or anti-GFP antibodies, respectively. **g** Acetylation level of HOXB13 was detected by a Co-IP assay using GFP-beads and an immunoblotting analysis with the anti-acetyl-lysine antibody in HEK293T cells. The cells were transiently transfected with GFP-HOXB13-WT along with the indicated plasmids or siRNAs. The protein levels of HBXIP and HOXB13 were separately examined by anti-Flag or anti-GFP antibodies. All experiments were repeated at least three times

To investigate the upregulation of HOXB13 mediated by HBXIP, we performed an immunoblotting analysis and found that HBXIP could stabilize HOXB13 under treatment with cycloheximide (CHX, a protein synthesis inhibitor) (Fig. 3a; Additional file 4: Figure S3a). Moreover, the overexpression of HBXIP was able to enhance the exogenous Flag-HOXB13 at the protein level (Fig. 3b; Additional file 4: Figure S3b), indicating that HBXIP might upregulate HOXB13 at a post-translational level. It has been reported that post-translational modification (PTM) can modulate the stability of proteins [35, 36]. According to PhosphoSitePlus (http://www.phosphosite.org/homeAction.action), there were two types of potential PTMs in HOXB13, including acetylation and phosphorylation. The Co-IP assay demonstrated that HOXB13 was acetylated and that its acetylation level increased with the overexpression of HBXIP (Fig. 3c; Additional file 4: Figure S3c). HOXB13 phosphorylation was not detected in this experiment. Furthermore, the protein levels of HOXB13 increased with trichostatin A (TSA, an inhibitor of deacetylase family HDACs) treatment, suggesting that the increase in acetylation enhances HOXB13 protein stability (Fig. 3d, e; Additional file 4: Figure S3d, e).

Next, we constructed Lys to Arg substitution mutants of the indicated sites (K270R, K277R, 270/277 double mutant, called DM). The acetylation levels of HOXB13 were no longer enhanced by HBXIP in the K277R and DM groups (Fig. 3f; Additional file 4: Figure S3f), implying that Lys277 was the HOXB13 acetylation site mediated by HBXIP. The sequence analysis revealed that Lys277 is evolutionarily conserved (Additional file 1: Figure S3g). It has been reported that HBXIP is capable of recruiting acetylases including p300 and GCN5, facilitating breast tumor progression [23, 37]. Notably, the knockdown of p300 effectively abolished the HBXIP-mediated increase of acetylation of HOXB13, while silencing GCN5 did not result in obvious change (Fig. 3g; Additional file 4: Figure S3h, i). Meanwhile, the Co-IP assay demonstrated that p300 could interact with HOXB13 (Additional file 4: Figure S3j). Silencing p300 impaired the HBXIP-mediated increase in HOXB13 in MCF-7 cells (Additional file 4: Figure S3k). These findings suggest that HBXIP enhances the acetylation of HOXB13 at the K277 site via the acetylase p300.

### HBXIP-enhanced acetylation of HOXB13 stabilizes HOXB13 in facilitation of TAM resistance

**Fig. 4:**
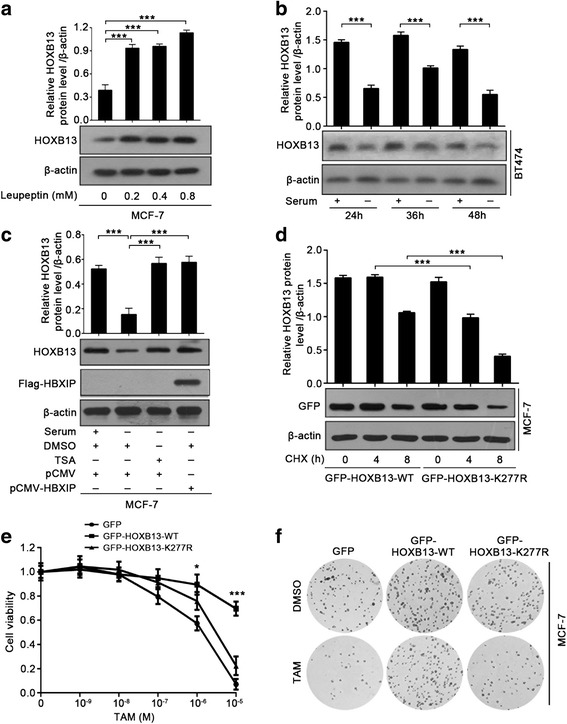
HBXIP-enhanced acetylation of HOXB13 stabilizes HOXB13 in the facilitation of TAM resistance. **a** Immunoblotting analysis of HOXB13 in MCF-7 cells treated with the indicated concentrations of leupeptin for 36 h (lower panel). The upper panel is the quantification of the intensity relative to β-actin. **b** Immunoblotting analysis of HOXB13 in BT474 cells cultured with serum-supplemented or serum-free media for the indicated time courses (lower panel). The upper panel is the quantification of the intensity relative to β-actin. **c** Immunoblotting analysis of HOXB13 in MCF-7 cells cultured with serum-supplemented or serum-free media for 48 h along with DMSO or TSA (1 μM) (lower panel). Before that, the cells were transiently transfected with pCMV or pCMV-HBXIP (1.5 μg). The protein level of HBXIP was determined by the anti-Flag antibody. The upper panel is the quantification of the intensity relative to β-actin. **d** Immunoblotting analysis of GFP-HOXB13 in MCF-7 cells time-dependently treated with 100 μg/ml CHX after being transiently transfected with GFP-HOXB13-WT or GFP-HOXB13-K277R (lower panel). The protein level of GFP-HOXB13 was determined by the anti-GFP antibody. The upper panel is the quantification of the intensity relative to β-actin. **e** Cell viability assay with MCF-7 cells treated with the indicated concentrations of TAM after being transiently transfected with the displayed plasmids. Error bars represent ± SD. *P < 0.05 and ***P < 0.001 (GFP-HOXB13-WT compared with GFP-HOXB13-K277R) by two-tailed Student’s *t* test. **f** A colony photograph of MCF-7 cells treated with DMSO or TAM (1 μM) after being transiently transfected with the displayed plasmids. All experiments were repeated at least three times. Error bars represent ± SD. *P < 0.05 and ***P < 0.001 by two-tailed Student’s *t* test

Next, we wanted to clarify the mechanism of HOXB13 stabilization that is mediated by HBXIP. The ubiquitin-proteasome pathway and the autophagy-lysosome pathway are the two main pathways for the degradation of intracellular proteins [35]. Treatment with MG132, a proteasome inhibitor, had no obvious impact on the HOXB13 protein level (Additional file 4: Figure S4a). However, treatment with leupeptin, an inhibitor of lysosomal proteases, induced a marked increase in the level of the HOXB13 protein (Fig. 4a). It is generally recognized that chaperone-mediated autophagy (CMA) selectively delivers proteins into lysosomes for degradation [38–40]. The substrates of CMA usually contain specific motifs and bind with heat shock 70 kDa protein 8 (HSC70), a molecular chaperone in CMA [41]. We discovered two similar lysosome-targeted motifs in the HOXB13 protein sequence, EPPKQ and QLREL (Additional file 4: Figure S4b). The Co-IP assay showed that HOXB13 interacted with HSC70 and that the interaction could be abolished by the overexpression of HBXIP (Additional file 4: Figure S4c). Cuervo’s group has reported that prolonged serum starvation can activate CMA in cells [42]. We cultured BT474 cells with serum starvation in different time courses and found that the protein level of HOXB13 was markedly reduced (Fig. 4b). However, the CMA-induced reduction of HOXB13 level could be rescued by TSA treatment or HBXIP overexpression (Fig. 4c). In addition, the immunoblotting analysis showed that the K277R mutant of HOXB13 was more easily degradable than the WT-HOXB13 (Fig. 4d). This evidence indicates that HBXIP-mediated acetylation of HOXB13 can protect HOXB13 from CMA-dependent degradation.

Then, we investigated the effect of HOXB13 acetylation mutation on TAM resistance. The cell viability and colony formation assays revealed that compared with WT-HOXB13, K277R-HOXB13 was unable to induce resistance to TAM in MCF-7 cells (Fig. 4e, f; Additional file 4: Figure S4d). Shah’s group has reported that IL-6 is a downstream target gene of HOXB13 in HOXB13-induced TAM resistance [43]. We further confirmed that the K277R and DM mutants of HOBX13 failed to increase IL-6 secretion, while the WT and the K270R mutant of HOXB13 effectively increased IL-6 secretion by ELISA (Additional file 4: Figure S4e). Thus, our data support that HBXIP-enhanced acetylation of HOXB13 stabilizes HOXB13 in the facilitation of TAM resistance.

### HBXIP co-activates HOXB13 to stimulate IL-6 transcription

**Fig. 5:**
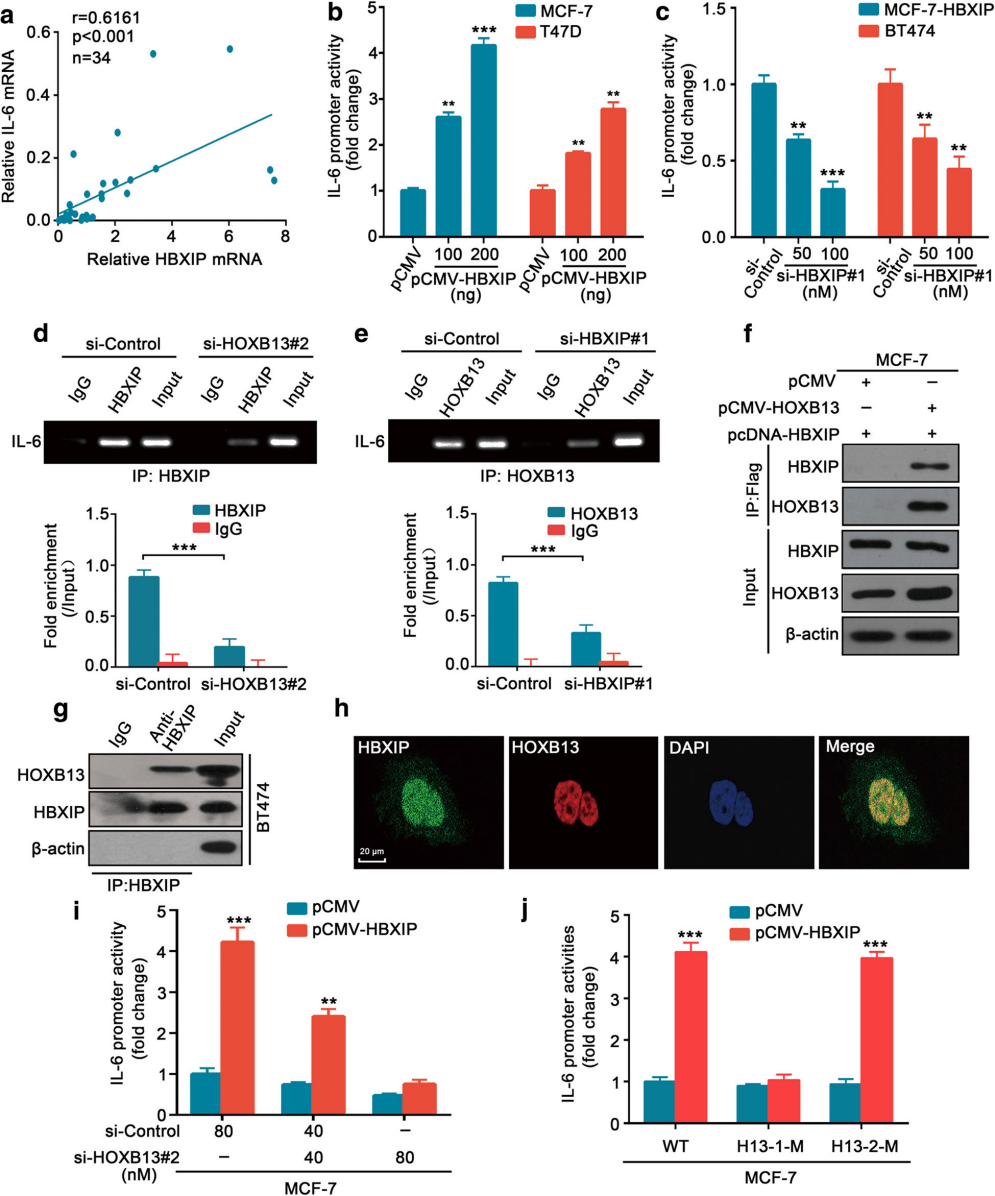
HBXIP co-activates HOXB13 to stimulate IL-6 transcription. **a** Relative mRNA levels of HBXIP and IL-6 in 34 ER+ clinical breast tumor tissues examined by qRT-PCR assay. The correlation between HBXIP and IL-6 mRNA levels was determined by Pearson’s correlation coefficient. **b**, **c** The luciferase reporter gene assay of IL-6 promoter activity in MCF-7 and T47D cells (**b**) and MCF-7-HBXIP and BT474 cells (**c**). The cells were transiently transfected with the indicated plasmids or siRNA. The luciferase activities were measured after transfection for 24 h. **d** ChIP assay in BT474 cells immunoprecipitated with anti-HBXIP antibody or control IgG after being transiently transfected with the si-control or si-HOXB13#2. The lower panel shows the quantitative enrichment data of the IL-6 promoter analyzed by qPCR and normalized against the input. **e** A similar assay as in **d** but immunoprecipitated with the anti-HOXB13 antibody or control IgG after being transiently transfected with the si-control or si-HBXIP#1. **f** Interaction of Flag-HOXB13 with HBXIP was analyzed by Co-IP assay in MCF-7 cells. The cells were transiently transfected with pCMV or pCMV-HOXB13 along with pcDNA-HBXIP. **g** Interaction of endogenous HBXIP with HOXB13 was examined by Co-IP assay in BT474 cells. **h** Co-localization of endogenous HBXIP and HOXB13 in BT474 cells was examined by confocal microscopy. Scale bar, 20 μm. **i** Luciferase reporter gene assay of IL-6 promoter activity in MCF-7 cells transiently transfected with pCMV or pCMV-HBXIP along with the indicated si-control or si-HOXB13#2. **j** Luciferase reporter gene assay of IL-6 promoter activities in MCF-7 cells. The cells were transiently transfected with pCMV or pCMV-HBXIP along with the IL-6 promoter (WT) or constructs with mutated binding sites of HOXB13-site1 (− 270/− 251, called H13-1-M) or HOXB13-site2 (− 135/− 121, called H13-2-M). All experiments were repeated at least three times. Error bars represent ± SD. **P < 0.01 and ***P < 0.001 by two-tailed Student’s *t* test

HOXB13, as a transcription factor, is capable of inhibiting ER-α and upregulating IL-6 expression in the promotion of TAM resistance [43]. Therefore, we investigated the impact of HBXIP on ER-α and IL-6. As expected, HBXIP markedly suppressed the expression of ER-α at the mRNA and protein levels (Additional file 4: Figure S5a). Meanwhile, the qRT-PCR assay showed that HBXIP could enhance the mRNA expression of IL-6 (Additional file 4: Figure S5b, c). Moreover, the mRNA levels of HBXIP were positively associated with those of IL-6 in 34 ER+ clinical breast cancer tissues (Fig. 5a). The luciferase reporter gene assay showed that HBXIP participated in the activation of IL-6 transcription (Fig. 5b, c). The ELISA revealed an increase in IL-6 secretion upon HBXIP overexpression in TAM-sensitive cells (Additional file 4: Figure S5d, e). The evidence reveals that HBXIP can function as a co-activator of the transcription factor to regulate gene expression [22]. Thus, we considered whether the co-activation function of HBXIP was involved in the modulation of IL-6. The ChIP assay showed that HBXIP could occupy the promoter of IL-6 (Fig. 5d). However, silencing HOXB13 impaired the interaction of HBXIP with the IL-6 promoter in BT474 cells, and vice versa (Fig. 5d, e). The Co-IP assay further demonstrated that HBXIP could interact with HOXB13 in MCF-7 and BT474 cells (Fig. 5f, g). Similarly, the co-localization of endogenous HBXIP with HOXB13 was revealed by confocal microscopic analysis (Fig. 5h). The knockdown of HOXB13 abolished HBXIP-increased promoter activity and secretion of IL-6 in MCF-7 and T47D cells (Fig. 5i; Additional file 4: Figure S5d–f). Meanwhile, the mutation of HOXB13-binding site1 (− 270/− 251) but not the binding site2 (− 135/− 121) in the IL-6 promoter significantly abrogated the HBXIP-mediated increase in promoter activity, indicating that HOXB13-site1 was responsible for the HBXIP-co-activated IL-6 transcription (Fig. 5j; Additional file 1: Figure S5g). In addition, cell viability and colony formation assays demonstrated that IL-6 blockade markedly impaired HBXIP-promoted cell proliferation and TAM resistance in MCF-7 cells (Additional file 4: Figure S5h, i). Thus, we conclude that HBXIP can co-activate HOXB13 to stimulate IL-6 transcription.

### ASA suppresses HBXIP/HOXB13 axis by reducing HBXIP expression

**Fig. 6:**
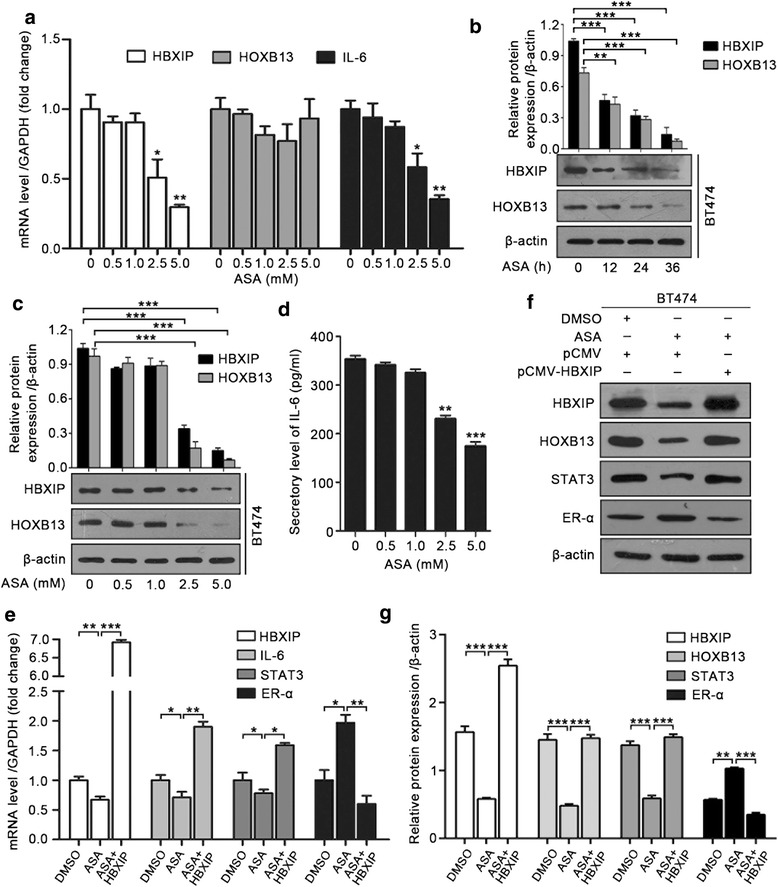
ASA suppresses HBXIP/HOXB13 axis by reducing HBXIP expression. **a** qRT-PCR assay of HBXIP, HOXB13, and IL-6 in BT474 cells treated with the displayed doses of ASA for 24 h. **b** Immunoblotting analysis of HBXIP and HOXB13 in BT474 cells treated with 2.5 mM ASA for the indicated time points (lower panel). The upper panel is the quantification of the intensity relative to β-actin. **c** Immunoblotting analysis of HBXIP and HOXB13 in BT474 cells treated with different concentrations of ASA for 24 h (lower panel). The upper panel is the quantification of the intensity relative to β-actin. **d** ELISA of IL-6 secretion in MCF-7-HBXIP cells treated with the indicated doses of ASA for 24 h. **e** qRT-PCR assay of HBXIP, IL-6, STAT3, and ER-α in BT474 cells treated with DMSO or ASA (2.5 mM) for 24 h after being transiently transfected with pCMV or pCMV-HBXIP. **f** Immunoblotting analysis of HBXIP, HOXB13, STAT3, and ER-α in BT474 cells treated with DMSO or ASA (2.5 mM) for 24 h after being transiently transfected with pCMV or pCMV-HBXIP. **g** The quantification of the intensity relative to β-actin in **f**. All experiments were repeated at least three times. Error bars represent ± SD. *P < 0.05, **P < 0.01, and ***P < 0.001 by two-tailed Student’s *t* test

We have found that HBXIP can serve as an upstream controller to activate some downstream effectors of ASA treatment including cyclooxygenase 2 (COX2), NF-kB, or extracellular signal-related kinase (ERK) [19, 29–31]. Here, we are wondering whether ASA is involved in HBXIP-associated TAM resistance. Thus, we examined the effect of ASA on the HBXIP/HOXB13 axis in breast cancer. Strikingly, ASA dose-dependently downregulated the expression of HBXIP, HOXB13, and IL-6 (Fig. 6a–d). STAT3, as a classic downstream effector of IL-6, can be transcriptionally upregulated by IL-6 [44]. The qRT-PCR assay and immunoblotting analysis showed that ASA could inhibit the expression of STAT3, as well as restore ER-α expression (Fig. 6e–g). However, overexpression of HBXIP abolished the ASA-induced inhibition of the HBXIP/HOXB13 axis (Fig. 6e–g).

Recent studies have revealed that ASA is capable of regulating the expression levels of microRNAs to affect tumor progression [45]. Previously, our group reported that miR-520b could directly target the 3′UTR of HBXIP mRNA [29]. Therefore, we considered whether ASA decreased HBXIP levels via miR-520b. Notably, ASA dose-dependently promoted the expression of miR-520b in BT474 cells (Additional file 4: Figure S6a). The qRT-PCR assay revealed the negative association between the expression of HBXIP and miR-520b, where miR-520b showed higher expression levels in TAM-sensitive MCF-7 and T47D cells but lower expression levels in MCF-7-HBXIP and TAM-resistant BT474 cells (Additional file 4: Figure S6b). Moreover, introducing the miR-520b inhibitor into BT474 cells achieved an impressive blocking effect on the ASA-mediated suppression of the HBXIP/HOXB13 axis (Additional file 4: Figure S6c, d). Taken together, our findings reveal that ASA inhibits the HBXIP/HOXB13 axis by reducing the expression of HBXIP.

### ASA-inhibited HBXIP/HOXB13 axis contributes to the reversal of TAM resistance

**Fig. 7:**
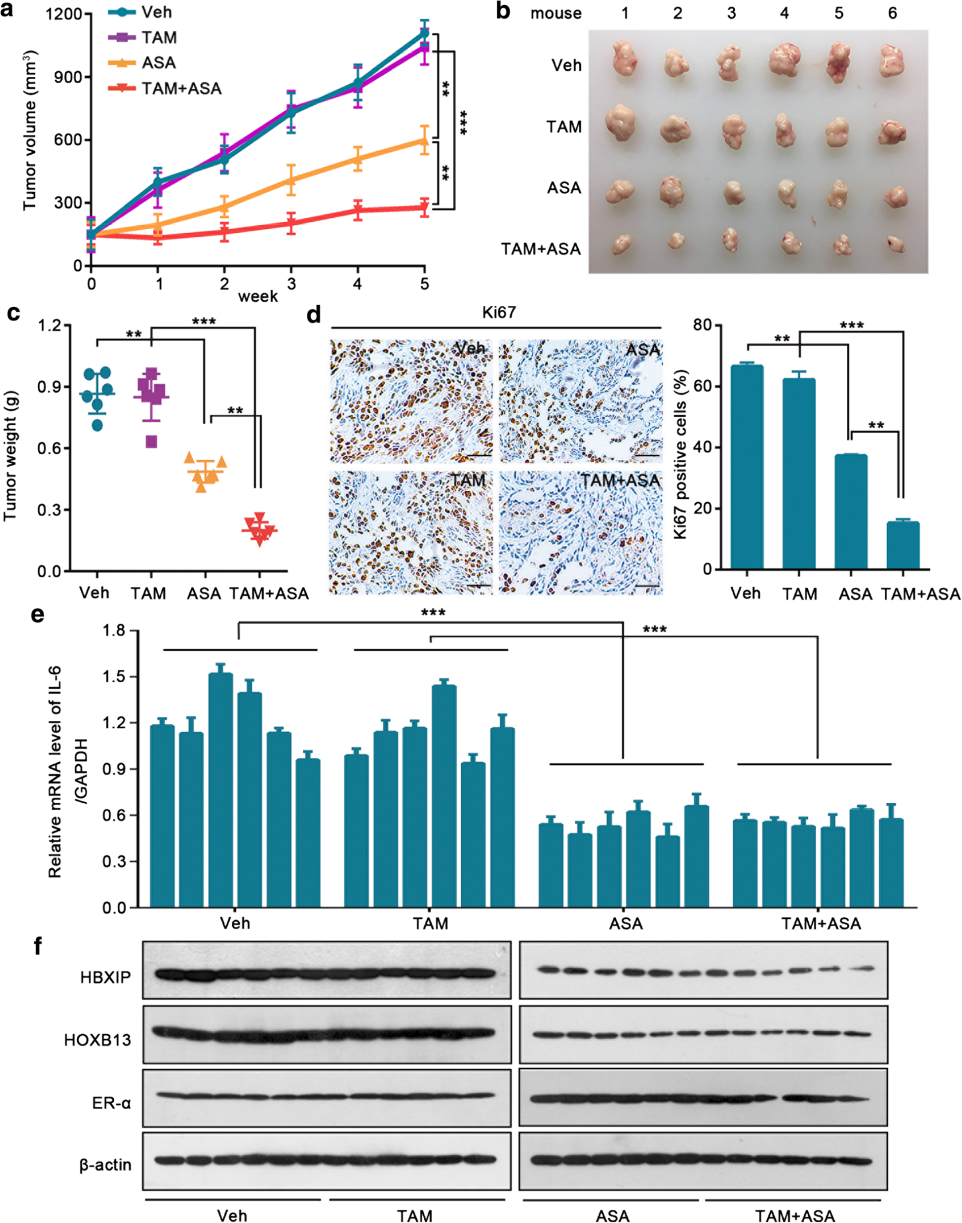
ASA-inhibited HBXIP/HOXB13 axis contributes to the reversal of TAM resistance. Growth curve (**a**) and imaging (**b**) of the xenograft tumors derived from BT474 cells with β-estradiol supplementation. After the tumors reached an approximate volume of 150 mm^3^, the mice were randomized into four treatment groups and were treated daily with the gavage administration of physiological saline (Veh), ASA (suspended in physiological saline, 75 mg/kg), TAM (suspended in physiological saline, 5 mg/kg), or a combination of ASA and TAM (TAM + ASA). **c** Weights of the xenograft tumors derived from BT474 cells shown in **a**. **d** Ki67 staining by IHC assay and the statistics of the Ki67-positive cells of the xenograft tumors derived from BT474 cells shown in **a**. Scale bar, 100 μm. **e** qRT-RCR assay of IL-6 expression in the xenograft tumors derived from BT474 cells shown in **a**. **f** Immunoblotting analysis of HBXIP, HOXB13, and ER-α in the xenograft tumors derived from BT474 cells shown in **a**. Error bars represent ± SD. **P < 0.01 and ***P < 0.001 by two-tailed Student’s *t* test

Next, we tested whether ASA could relieve TAM resistance by depressing the HBXIP/HOXB13 axis. The cell viability analysis showed that the combination of ASA with TAM achieved a more effective inhibition of breast cancer cell growth than TAM alone (Additional file 4: Figure S7a, b). The colony formation assay showed a similar result in BT474 cells (Additional file 4: Figure S7c, d). mfp xenografts using BT474 cells demonstrated that TAM combined with ASA markedly inhibited tumor growth, in contrast to the use of TAM or ASA alone (Fig. 7a–c). No difference was observed in the baseline body weights among the four groups, indicating that the mice remained healthy throughout the above treatment schedule (Additional file 4: Figure S7e). Moreover, the expression of Ki67 in the combination group significantly decreased relative to the expression in the groups administered only TAM or ASA (Fig. 7d). The qRT-PCR assay and immunoblotting analysis demonstrated that the expression levels of HBXIP, HOXB13, IL-6, and ER-α in each group of mice tumor samples were consistent with the aforesaid results (Fig. 7e, f; Additional file 4: Figure S7f–h). Taken together, our findings reveal that the ASA-inhibited HBXIP/HOXB13 axis contributes to the reversal of TAM resistance in vitro and in vivo.

## Discussion

Drug resistance frequently occurs in clinical therapy, becoming a stumbling block in targeted treatment of cancer [46]. Although tamoxifen (TAM) initially achieves a favorable response in the inhibition of ER+ breast cancer progression, the inherent or developed resistance to it inevitably appears [47, 48]. It is important to identify the biomarkers predicting response to TAM so that an alternative therapeutic regimen can be used. Studies have demonstrated the significance of HOXB13:IL17BR index in predicting TAM resistance [10, 11]. Further study unveils that HOXB13 alone can be used for the prediction of TAM resistance [14]. HOXB13 is not only a predictor of TAM resistance but also a marker of resistance to endocrine therapy per se [15]. A higher HOXB13 expression in ER+ breast tumors always renders patients less likely to respond to TAM [14, 43]. Ma et al. uncover that HOXB13 can be used to predict response to the treatment of letrozole (an aromatase inhibitor) [49].

Studying TAM resistance in breast cancer is of potential high significance; however, the approaches applied in the identification of biomarker are not always unbiased. As reviews published on journals of Nat Rev. Clin Oncol and J Natl Cancer Inst described [50, 51], the way to distinguish a predictive biomarker from a prognostic biomarker is analyzing tumor samples of patients participated in a randomized controlled trial (RCT), which would enable testing for an interaction between the proposed predictive biomarker and the treatment. For instance, if a given biomarker does not affect prognosis, the presence or absence of this biomarker will have similar prognoses without treatment. In patients with the biomarker, treatment with TAM will not change their outcome, but patients without the biomarker will benefit from TAM treatment. In this case, the biomarker can be used for predicting TAM resistance. Although studies have reported that the HOXB13:IL17BR index can predict clinical outcome of TAM monotherapy [10–13], HOXB13:IL17BR index and the independent roles of HOXB13 and IL17BR were not be evaluated in a randomized study comparing TAM vs. no TAM treatment until 2008 [14].

Much is disclosed about the important role of HOXB13 in the prediction of TAM therapy, the underlying mechanism of HOXB13 expression regulation in TAM resistance of breast cancer is largely unclear. As an oncoprotein, HBXIP can affect cell proliferation, migration, angiogenesis, and aberrant lipid metabolism to promote the development of breast cancer [20, 21, 24, 52, 53]. In this study, we are interested in whether HBXIP is involved in TAM resistance of breast cancer. The analysis of Kaplan-Meier plotter firstly revealed that HBXIP expression was inversely associated with the relapse-free survival of TAM-treated breast cancer patients [32]. Our function analysis confirmed the role of HBXIP in the promotion of TAM resistance. As Beelen et al. and Coleman et al. summarized, another issue that should be aware in biomarker identification is the lack of differentiation between premenopausal and post-menopausal patients with breast cancer [50, 54]. The hormonal environment of a tumor arising in a premenopausal patient is intrinsically distinct from one arising in a post-menopausal patient. In our present study, we used the Kaplan-Meier plotter online resource to show the relationship of HBXIP and relapse-free survival of mono-TAM-treated patients. However, the information about whether the patients are premenopausal or post-menopausal is not easy to obtain in the Kaplan-Meier plotter online resource.

In the next investigation, we evaluated the association of HBXIP with HOXB13 in TAM resistance. A positive correlation between HBXIP and HOXB13 was shown using Pearson chi-square independence test of a tissue microarray staining. Taken a step further, we demonstrated for the first time that the oncoprotein HBXIP could upregulate HOXB13 at the post-translational level, promoting TAM resistance. As a common PTM, lysine acetylation has been reported to regulate protein stability [55, 56]. Geng et al. indicate that acetylase p300 can acetylate HIF-1α and protect it from ubiquitin-mediated proteasomal degradation [56]. Here, we provided evidence that p300 recruited by HBXIP was required for the K277 acetylation of HOXB13 in the maintenance of HOXB13 stability. Several studies have demonstrated that the CMA pathway can degrade transcription factors and nuclear receptor co-repressors, such as N-COR and MEF2A [57, 58]. In this study, we discovered that HBXIP-mediated enhancement of HOXB13 acetylation efficiently protected HOXB13 from CMA-mediated degradation, leading to the accumulation of HOXB13.

The main cause of TAM resistance is the occurrence of the abnormal-activated ER-α pathway or alternative pro-proliferative pathway after turning off ER-α pathway [47, 59–65]. HOXB13 confers TAM resistance by reducing the expression of ER-α and inducing IL-6 expression [43]. Our finding demonstrated that HBXIP could inhibit the expression of ER-α, possibly through the HOXB13-mediated inhibition of ER-α transcription. The activation of a substitutive pro-proliferative pathway is a pivotal part in TAM resistance. We found that HBXIP participated in HOXB13-stimulated IL-6 transcription by co-activating HOXB13, leading to the promotion of cell proliferation. Thus, HBXIP/HOXB13 axis efficiently circumvents TAM by converting ER-α-dependent cell growth to IL-6-dependent, providing alternative proliferative stimuli for tumor cells and making the cells continue to grow.

Aspirin (ASA), a classic anti-inflammatory agent, has been reported to play novel roles in the reduction of incidences of cancers including breast cancer [26–28]. ASA treatment has many downstream, such as cyclooxygenase 2 (COX2), NF-kB, extracellular signal-related kinase (ERK), or some lncRNAs and miRNAs [45, 66–69]. Our previous study has demonstrated that HBXIP can activate NF-kB and ERK [19, 29, 31]. Another report shows that pERK1/2 can upregulate COX2 expression for maintaining proliferation and migration of breast cancer [30], indicating that COX2 could be a downstream effector of HBXIP in cancers. Thus, we chose ASA to tackle HBXIP-mediated TAM resistance in this study. As our data showed, ASA relieved TAM resistance by inhibiting HBXIP. Our finding is consistent with a recent study in which ASA has a potential role in overcoming TAM resistance [70]. Previously, our group reported that miR-520b could directly target HBXIP in breast cancer [29]. Many studies have demonstrated that salicylates and other NSAIDs can upregulate tumor suppressor-type miRNAs to inhibit tumor progression. [45, 71]. Here, we uncovered that ASA could suppress HBXIP via inducing miR-520b, a tumor suppressor miRNA, but the regulation mechanism of miR-520b mediated by ASA needs more investigation in the future. Oncoprotein HBXIP promotes breast cancer by regulating different cancer-related proteins [20, 21, 23, 24]. We observed that treatment with ASA could result in marked suppression of breast cancer growth, which might due to the inhibition of HBXIP-promoted breast cancer progression. It is therapeutically meaningful to combine ASA with TAM to treat ER+ breast cancer with high HBXIP expression levels.

## Conclusion

In summary, our study discloses a new mechanism for provoking HOXB13-associated TAM resistance (Additional file 1: Figure S8). In this model, HBXIP can prevent CMA-dependent degradation of HOXB13 by increasing its acetylation at K277 via acetylase p300, resulting in the accumulation of HOXB13. Moreover, HBXIP inhibits the expression of ER-α by HOXB13, leading to TAM off-target, and acts as a co-activator of HOXB13 to stimulate IL-6 transcription, resulting in the acceleration of proliferation. The ASA-mediated upregulation of miR-520b suppresses HBXIP expression and then blocks the HBXIP/HOXB13 axis, overcoming TAM resistance. Our finding provides insights into the mechanism by which the oncoprotein HBXIP modulates HOXB13 to confer TAM resistance. Therapeutically, ASA can serve as an effective agent for relieving TAM resistance by inhibiting HBXIP expression.

## Additional files

Additional file 1: **Table S1.** List of primers and siRNA sequences used in this paper. (DOCX 22 kb)

Additional file 2: **Table S2.** Clinical characteristics of breast tissue microarray. (DOC 165 kb)

Additional file 3: **Table S3.** Clinical characteristics of 34 ER+ breast cancer tissue samples. (DOCX 19 kb)

Additional file 4: **Figure S1.** HBXIP contributes to TAM resistance in breast cancer. **Figure S2.** HBXIP induces TAM resistance by increasing the protein level of HOXB13. **Figure S3.** HBXIP enhances acetylation of HOXB13 at K277 site via acetylase p300. **Figure S4.** HBXIP-enhanced acetylation of HOXB13 stabilizes HOXB13 in the facilitation of TAM resistance. **Figure S5.** HBXIP co-activates HOXB13 to stimulate IL-6 transcription. **Figure S6.** ASA suppresses HBXIP/HOXB13 axis by reducing HBXIP expression. **Figure S7.** ASA-inhibited HBXIP/HOXB13 axis contributes to the reversal of TAM resistance. **Figure S8.** Diagram of working model. (ZIP 1058 kb)

Additional file 5: Additional methods. (DOCX 27 kb)

## References

[1] Vargo-Gogola T, Rosen JM (2007). Modelling breast cancer: one size does not fit all. Nat Rev Cancer.

[2] Piva M, Domenici G, Iriondo O, Rabano M, Simoes BM, Comaills V, Barredo I, Lopez-Ruiz JA, Zabalza I, Kypta R, Vivanco M (2014). Sox2 promotes tamoxifen resistance in breast cancer cells. EMBO Mol Med.

[3] Jaiyesimi IA, Buzdar AU, Decker DA, Hortobagyi GN (1995). Use of tamoxifen for breast cancer: twenty-eight years later. J Clin Oncol.

[4] Connor CE, Norris JD, Broadwater G, Willson TM, Gottardis MM, Dewhirst MW, McDonnell DP (2001). Circumventing tamoxifen resistance in breast cancers using antiestrogens that induce unique conformational changes in the estrogen receptor. Cancer Res.

[5] Bhatt S, Stender JD, Joshi S, Wu G, Katzenellenbogen BS (2016). OCT-4: a novel estrogen receptor-alpha collaborator that promotes tamoxifen resistance in breast cancer cells. Oncogene.

[6] Massarweh S, Schiff R (2007). Unraveling the mechanisms of endocrine resistance in breast cancer: new therapeutic opportunities. Clin Cancer Res.

[7] Droog M, Beelen K, Linn S, Zwart W (2013). Tamoxifen resistance: from bench to bedside. Eur J Pharmacol.

[8] Miao J, Wang Z, Provencher H, Muir B, Dahiya S, Carney E, Leong CO, Sgroi DC, Orsulic S (2007). HOXB13 promotes ovarian cancer progression. Proc Natl Acad Sci U S A.

[9] Kim YR, Kim IJ, Kang TW, Choi C, Kim KK, Kim MS, Nam KI, Jung C (2014). HOXB13 downregulates intracellular zinc and increases NF-kappaB signaling to promote prostate cancer metastasis. Oncogene.

[10] Ma XJ, Wang Z, Ryan PD, Isakoff SJ, Barmettler A, Fuller A, Muir B, Mohapatra G, Salunga R, Tuggle JT (2004). A two-gene expression ratio predicts clinical outcome in breast cancer patients treated with tamoxifen. Cancer Cell.

[11] Jansen MP, Sieuwerts AM, Look MP, Ritstier K, Meijer-van Gelder ME, van Staveren IL, Klijn JG, Foekens JA, Berns EM (2007). HOXB13-to-IL17BR expression ratio is related with tumor aggressiveness and response to tamoxifen of recurrent breast cancer: a retrospective study. J Clin Oncol.

[12] Ma XJ, Hilsenbeck SG, Wang W, Ding L, Sgroi DC, Bender RA, Osborne CK, Allred DC, Erlander MG (2006). The HOXB13:IL17BR expression index is a prognostic factor in early-stage breast cancer. J Clin Oncol.

[13] Goetz MP, Suman VJ, Couch FJ, Ames MM, Rae JM, Erlander MG, Ma XJ, Sgroi DC, Reynolds CA, Lingle WL (2008). Cytochrome P450 2D6 and homeobox 13/interleukin-17B receptor: combining inherited and tumor gene markers for prediction of tamoxifen resistance. Clin Cancer Res.

[14] Jerevall PL, Brommesson S, Strand C, Gruvberger-Saal S, Malmstrom P, Nordenskjold B, Wingren S, Soderkvist P, Ferno M, Stal O (2008). Exploring the two-gene ratio in breast cancer--independent roles for HOXB13 and IL17BR in prediction of clinical outcome. Breast Cancer Res Treat.

[15] Jerevall PL, Jansson A, Fornander T, Skoog L, Nordenskjold B, Stal O (2010). Predictive relevance of HOXB13 protein expression for tamoxifen benefit in breast cancer. Breast Cancer Res.

[16] Bar-Peled L, Schweitzer LD, Zoncu R, Sabatini DM (2012). Ragulator is a GEF for the rag GTPases that signal amino acid levels to mTORC1. Cell.

[17] Fujii R, Zhu C, Wen Y, Marusawa H, Bailly-Maitre B, Matsuzawa S, Zhang H, Kim Y, Bennett CF, Jiang W, Reed JC (2006). HBXIP, cellular target of hepatitis B virus oncoprotein, is a regulator of centrosome dynamics and cytokinesis. Cancer Res.

[18] Melegari M, Scaglioni PP, Wands JR (1998). Cloning and characterization of a novel hepatitis B virus x binding protein that inhibits viral replication. J Virol.

[19] Li Y, Zhang Z, Zhou X, Li L, Liu Q, Wang Z, Bai X, Zhao Y, Shi H, Zhang X, Ye L (2014). The oncoprotein HBXIP enhances migration of breast cancer cells through increasing filopodia formation involving MEKK2/ERK1/2/Capn4 signaling. Cancer Lett.

[20] Li L, Fang R, Liu B, Shi H, Wang Y, Zhang W, Zhang X, Ye L (2016). Deacetylation of tumor-suppressor MST1 in Hippo pathway induces its degradation through HBXIP-elevated HDAC6 in promotion of breast cancer growth. Oncogene.

[21] Zhao Y, Li H, Zhang Y, Li L, Fang R, Li Y, Liu Q, Zhang W, Qiu L, Liu F (2016). Oncoprotein HBXIP modulates abnormal lipid metabolism and growth of breast cancer cells by activating the LXRs/SREBP-1c/FAS signaling cascade. Cancer Res.

[22] Wang Y, Fang R, Cui M, Zhang W, Bai X, Wang H, Liu B, Zhang X, Ye L (2017). The oncoprotein HBXIP up-regulates YAP through activation of transcription factor c-Myb to promote growth of liver cancer. Cancer Lett.

[23] Li H, Liu Q, Wang Z, Fang R, Shen Y, Cai X, Gao Y, Li Y, Zhang X, Ye L (2015). The oncoprotein HBXIP modulates the feedback loop of MDM2/p53 to enhance the growth of breast cancer. J Biol Chem.

[24] Li Y, Wang Z, Shi H, Li H, Li L, Fang R, Cai X, Liu B, Zhang X, Ye L (2016). HBXIP and LSD1 scaffolded by lncRNA Hotair Mediate Transcriptional Activation by c-Myc. Cancer Res.

[25] Khuder SA, Mutgi AB (2001). Breast cancer and NSAID use: a meta-analysis. Br J Cancer.

[26] Terry MB, Gammon MD, Zhang FF, Tawfik H, Teitelbaum SL, Britton JA, Subbaramaiah K, Dannenberg AJ, Neugut AI (2004). Association of frequency and duration of aspirin use and hormone receptor status with breast cancer risk. JAMA.

[27] Drew DA, Cao Y, Chan AT (2016). Aspirin and colorectal cancer: the promise of precision chemoprevention. Nat Rev Cancer.

[28] Sitia G, Aiolfi R, Di Lucia P, Mainetti M, Fiocchi A, Mingozzi F, Esposito A, Ruggeri ZM, Chisari FV, Iannacone M, Guidotti LG (2012). Antiplatelet therapy prevents hepatocellular carcinoma and improves survival in a mouse model of chronic hepatitis B. Proc Natl Acad Sci U S A.

[29] Hu N, Zhang J, Cui W, Kong G, Zhang S, Yue L, Bai X, Zhang Z, Zhang W, Zhang X, Ye L (2011). miR-520b regulates migration of breast cancer cells by targeting hepatitis B X-interacting protein and interleukin-8. J Biol Chem.

[30] You J, Mi D, Zhou X, Qiao L, Zhang H, Zhang X, Ye L (2009). A positive feedback between activated extracellularly regulated kinase and cyclooxygenase/lipoxygenase maintains proliferation and migration of breast cancer cells. Endocrinology.

[31] Cui W, Zhao Y, Shan C, Kong G, Hu N, Zhang Y, Zhang S, Zhang W, Zhang Y, Zhang X, Ye L (2012). HBXIP upregulates CD46, CD55 and CD59 through ERK1/2/NF-kappaB signaling to protect breast cancer cells from complement attack. FEBS Lett.

[32] Gyorffy B, Lanczky A, Eklund AC, Denkert C, Budczies J, Li Q, Szallasi Z (2010). An online survival analysis tool to rapidly assess the effect of 22, 277 genes on breast cancer prognosis using microarray data of 1, 809 patients. Breast Cancer Res Treat.

[33] Pawitan Y, Bjohle J, Amler L, Borg AL, Egyhazi S, Hall P, Han X, Holmberg L, Huang F, Klaar S (2005). Gene expression profiling spares early breast cancer patients from adjuvant therapy: derived and validated in two population-based cohorts. Breast Cancer Res.

[34] Ringner M, Fredlund E, Hakkinen J, Borg A, Staaf J (2011). GOBO: gene expression-based outcome for breast cancer online. PLoS One.

[35] Lv L, Li D, Zhao D, Lin R, Chu Y, Zhang H, Zha Z, Liu Y, Li Z, Xu Y (2011). Acetylation targets the M2 isoform of pyruvate kinase for degradation through chaperone-mediated autophagy and promotes tumor growth. Mol Cell.

[36] Inuzuka H, Gao D, Finley LW, Yang W, Wan L, Fukushima H, Chin YR, Zhai B, Shaik S, Lau AW (2012). Acetylation-dependent regulation of Skp2 function. Cell.

[37] Li L, Liu B, Zhang X, Ye L (2015). The oncoprotein HBXIP promotes migration of breast cancer cells via GCN5-mediated microtubule acetylation. Biochem Biophys Res Commun.

[38] Mizushima N, Levine B, Cuervo AM, Klionsky DJ (2008). Autophagy fights disease through cellular self-digestion. Nature.

[39] Yorimitsu T, Klionsky DJ (2005). Autophagy: molecular machinery for self-eating. Cell Death Differ.

[40] Shaid S, Brandts CH, Serve H, Dikic I (2013). Ubiquitination and selective autophagy. Cell Death Differ.

[41] Dice JF (2007). Chaperone-mediated autophagy. Autophagy.

[42] Cuervo AM, Knecht E, Terlecky SR, Dice JF (1995). Activation of a selective pathway of lysosomal proteolysis in rat liver by prolonged starvation. Am J Phys.

[43] Shah N, Jin K, Cruz LA, Park S, Sadik H, Cho S, Goswami CP, Nakshatri H, Gupta R, Chang HY (2013). HOXB13 mediates tamoxifen resistance and invasiveness in human breast cancer by suppressing ERalpha and inducing IL-6 expression. Cancer Res.

[44] Qi QR, Yang ZM (2014). Regulation and function of signal transducer and activator of transcription 3. World J Biol Chem.

[45] Yiannakopoulou E (2014). Targeting epigenetic mechanisms and microRNAs by aspirin and other non steroidal anti-inflammatory agents—implications for cancer treatment and chemoprevention. Cell Oncol (Dordr).

[46] Garraway LA, Janne PA (2012). Circumventing cancer drug resistance in the era of personalized medicine. Cancer Discov.

[47] Musgrove EA, Sutherland RL (2009). Biological determinants of endocrine resistance in breast cancer. Nat Rev Cancer.

[48] Wong PP, Yeoh CC, Ahmad AS, Chelala C, Gillett C, Speirs V, Jones JL, Hurst HC (2014). Identification of MAGEA antigens as causal players in the development of tamoxifen-resistant breast cancer. Oncogene.

[49] Ma X, Bandaru R, Letzkus M, Philips P, Barrett J, Erlander M, Goetz M, Sgroi D, Gardner H, Baselga J (2009). HOXB13 may predict response to neoadjuvant letrozole in patients with estrogen receptor-positive breast cancer. Cancer Res.

[50] Beelen K, Zwart W, Linn SC (2012). Can predictive biomarkers in breast cancer guide adjuvant endocrine therapy?. Nat Rev Clin Oncol.

[51] Simon RM, Paik S, Hayes DF (2009). Use of archived specimens in evaluation of prognostic and predictive biomarkers. J Natl Cancer Inst.

[52] Liu F, You X, Wang Y, Liu Q, Liu Y, Zhang S, Chen L, Zhang X, Ye L (2014). The oncoprotein HBXIP enhances angiogenesis and growth of breast cancer through modulating FGF8 and VEGF. Carcinogenesis.

[53] Liu S, Li L, Zhang Y, Zhang Y, Zhao Y, You X, Lin Z, Zhang X, Ye L (2012). The oncoprotein HBXIP uses two pathways to up-regulate S100A4 in promotion of growth and migration of breast cancer cells. J Biol Chem.

[54] Coleman RE (2011). Bone cancer in 2011: prevention and treatment of bone metastases. Nat Rev Clin Oncol.

[55] Zhao D, Zou SW, Liu Y, Zhou X, Mo Y, Wang P, Xu YH, Dong B, Xiong Y, Lei QY, Guan KL (2013). Lysine-5 acetylation negatively regulates lactate dehydrogenase A and is decreased in pancreatic cancer. Cancer Cell.

[56] Geng H, Liu Q, Xue C, David LL, Beer TM, Thomas GV, Dai MS, Qian DZ (2012). HIF1alpha protein stability is increased by acetylation at lysine 709. J Biol Chem.

[57] Ali AB, Nin DS, Tam J, Khan M (2011). Role of chaperone mediated autophagy (CMA) in the degradation of misfolded N-CoR protein in non-small cell lung cancer (NSCLC) cells. PLoS One.

[58] Zhang L, Sun Y, Fei M, Tan C, Wu J, Zheng J, Tang J, Sun W, Lv Z, Bao J (2014). Disruption of chaperone-mediated autophagy-dependent degradation of MEF2A by oxidative stress-induced lysosome destabilization. Autophagy.

[59] Osborne CK, Schiff R (2011). Mechanisms of endocrine resistance in breast cancer. Annu Rev Med.

[60] Clarke R, Tyson JJ, Dixon JM (2015). Endocrine resistance in breast cancer—an overview and update. Mol Cell Endocrinol.

[61] Feng Q, Zhang Z, Shea MJ, Creighton CJ, Coarfa C, Hilsenbeck SG, Lanz R, He B, Wang L, Fu X (2014). An epigenomic approach to therapy for tamoxifen-resistant breast cancer. Cell Res.

[62] Toy W, Shen Y, Won H, Green B, Sakr RA, Will M, Li Z, Gala K, Fanning S, King TA (2013). ESR1 ligand-binding domain mutations in hormone-resistant breast cancer. Nat Genet.

[63] Merenbakh-Lamin K, Ben-Baruch N, Yeheskel A, Dvir A, Soussan-Gutman L, Jeselsohn R, Yelensky R, Brown M, Miller VA, Sarid D (2013). D538G mutation in estrogen receptor-alpha: a novel mechanism for acquired endocrine resistance in breast cancer. Cancer Res.

[64] Jeselsohn R, Yelensky R, Buchwalter G, Frampton G, Meric-Bernstam F, Gonzalez-Angulo AM, Ferrer-Lozano J, Perez-Fidalgo JA, Cristofanilli M, Gomez H (2014). Emergence of constitutively active estrogen receptor-alpha mutations in pretreated advanced estrogen receptor-positive breast cancer. Clin Cancer Res.

[65] Dees EC, Carey LA (2013). Improving endocrine therapy for breast cancer: it’s not that simple. J Clin Oncol.

[66] Howard PA, Delafontaine P (2004). Nonsteroidal anti-inflammatory drugs and cardiovascular risk. J Am Coll Cardiol.

[67] Pillinger MH, Capodici C, Rosenthal P, Kheterpal N, Hanft S, Philips MR, Weissmann G (1998). Modes of action of aspirin-like drugs: salicylates inhibit erk activation and integrin-dependent neutrophil adhesion. Proc Natl Acad Sci USA.

[68] Amann R, Peskar BA (2002). Anti-inflammatory effects of aspirin and sodium salicylate. Eur J Pharmacol.

[69] Guo H, Liu J, Ben Q, Qu Y, Li M, Wang Y, Chen W, Zhang J (2016). The aspirin-induced long non-coding RNA OLA1P2 blocks phosphorylated STAT3 homodimer formation. Genome Biol.

[70] Cheng R, Liu YJ, Cui JW, Yang M, Liu XL, Li P, Wang Z, Zhu LZ, Lu SY, Zou L (2017). Aspirin regulation of c-myc and cyclinD1 proteins to overcome tamoxifen resistance in estrogen receptor-positive breast cancer cells. Oncotarget.

[71] Saito Y, Suzuki H, Imaeda H, Matsuzaki J, Hirata K, Tsugawa H, Hibino S, Kanai Y, Saito H, Hibi T (2013). The tumor suppressor microRNA-29c is downregulated and restored by celecoxib in human gastric cancer cells. Int J Cancer.

