# Supplementary material for: Oncoprotein HBXIP enhances HOXB13 acetylation and co-activates HOXB13 to confer tamoxifen resistance in breast cancer
Source: J Hematol Oncol. 2018 Feb 23;11:26. doi: 10.1186/s13045-018-0577-5 (PMC5824486; doi:10.1186/s13045-018-0577-5)
Supplement: Supplementary file 1 — Table S1. List of primers and siRNA sequences used in this paper. (DOCX 22 kb) [file 13045_2018_577_MOESM1_ESM.docx]

**Table S1** List of primers and siRNA sequences used in this paper.

| **Gene** | **Primer** | **Sequence (5′-3′)** |
| --- | --- | --- |
|  | | |
| **Primers for RT-PCR and qRT-PCR** | | |
| HBXIP | forward | CTTGGAGCAGCACTTGGAAGA |
|  | reverse | ATGCCATCGTGTTTCTGGATC |
| GAPDH | forward | AACGGATTTGGTCGTATTG |
|  | reverse | GGAAGATGGTGATGGGATT |
| HOXB13 | forward | GTGCTGCCCGCTGGAGTC |
|  | reverse | AGTTACCTGGACGTGTCTGTGG |
| IL-6 | forward | GAAAGCAGCAAAGAGGCACT |
|  | reverse | TTTCACCAGGCAAGTCTCCT |
| STAT3 | forward | TCAGTGACCAGGCAGAAGAT |
|  | reverse | TTGTTGACGGGTCTGAAGTT |
| ER-α | forward | CATGAAGTGCAAGAACGTGGTG |
|  | reverse | AAGGAATGCGATGAAGTAGAGCC |
| miR-520b | forward | AAAGTGCTTCCTTTTAGAGCG |
|  | reverse | GCGAGCACAGAATTAATACGAC |
| U6 | forward | CTCGCTTCGGCAGCACA |
|  | reverse | AACGCTTCACGAATTTGCGT |
| **Primers for ChIP** |  |  |
| IL-6 promoter | forward | AGACATGCCAAAGTGCTGA |
|  | reverse | GGGCTGATTGGAAACCTTAT |
| **Primers for IL-6**  **promoter fragments** |  |  |
| pGL-IL-6 | forward | GGGGTACCCTTAGCAAAGCAAAGAAACCG |
|  | reverse | CCCAAGCTTAATGAGCCTCAGACATCTCCAG |
| H13-1-M | forward | GTGCTGAGTCACACAGCGAAGAAA |
|  | reverse | TTTCTTCGCTGTGTGACTCAGCAC |
| H13-2-M | forward | GCACAATCTTACGAAGGTTTCCAAT |
|  | reverse | ATTGGAAACCTTCGTAAGATTGTGC |
| **Primers for full-length HOXB13** |  |  |
| pCMV-Tag2B-HOXB13 | forward | CCCAAGCTTATGGAGCCCGGCAATTATGCCA |
|  | reverse | CCGCTCGAGTTAAGGGGTAGCGCTGTTCTTC |
| pEGFP-C2-HOXB13 | forward | CCGGAATTCATGGAGCCCGGCAATTATGCCA |
|  | reverse | CGCGGATCCTTAAGGGGTAGCGCTGTTCTTC |
| K270R | forward | GAACCGCCGGGTCCGAGAGAAGAAGGTTCT |
|  | reverse | AGAACCTTCTTCTCTCGGACCCGGCGGTTC |
| K277R | forward | AGAAGGTTCTCGCCCGGGTGAAGAACAGCG |
|  | reverse | CGCTGTTCTTCACCCGGGCGAGAACCTTCT |
| **siRNA duplexes** |  |  |
| Control siRNA | sense | UUCUCCGAACGUGUCACGUdTdT |
| HBXIP siRNA #1 | sense | CGGAAGCGCAGUGAUGUUUdTdT |
| HBXIP siRNA #2  HOXB13 siRNA #1 | sense  sense | GCAGCUAAGGCAGCUAAGCUAACCUCUGdTdT  CCUUGCAUACUUAGCCCUUdTdT |
| HOXB13 siRNA #2 | sense | CGCCAGAUUACCAUCUGGTdTdT |
| p300 siRNA #1 | sense | CUAGAGACACCUUGUAGUAdTdT |
| p300 siRNA #2 | sense | AACAGAGCAGUCCUGGAUUAGdTdT |
| GCN5 siRNA #1 | sense | UGUUCGAGCUCUCAAAGAUdTdT |
| GCN5 siRNA #2 | sense | GGAAAUGCAUCCUGCAGAUdTdT |
|  |  |  |
