# Supplementary material for: Oncoprotein HBXIP enhances HOXB13 acetylation and co-activates HOXB13 to confer tamoxifen resistance in breast cancer
Source: J Hematol Oncol. 2018 Feb 23;11:26. doi: 10.1186/s13045-018-0577-5 (PMC5824486; doi:10.1186/s13045-018-0577-5)
Supplement: Supplementary file 2 — Table S2. Clinical characteristics of breast tissue microarray. (DOC 165 kb) [file 13045_2018_577_MOESM2_ESM.doc]

**Table S2**. Clinical characteristics of breast tissue microarray containing breast carcinoma, Lymph node metastatic carcinoma, different breast diseases and normal breast tissue samples

| **No.** | **Age** | **Sex** | **Organ** | **Pathology diagnosis** | **Grade** | **ER** |
| --- | --- | --- | --- | --- | --- | --- |
| 01 | 55 | F | Breast | Lymph node metastatic infiltrating ductal carcinoma | I | + |
| 02 | 56 | F | Breast | Lymph node metastatic infiltrating ductal carcinoma | II | +++ |
| 03 | 48 | F | Breast | Lymph node metastatic infiltrating ductal carcinoma | II | + |
| 04 | 38 | F | Breast | Lymph node metastatic infiltrating ductal carcinoma | II | ++ |
| 05 | 44 | F | Breast | Lymph node metastatic infiltrating ductal carcinoma | II | +++ |
| 06 | 56 | F | Breast | Lymph node metastatic infiltrating ductal carcinoma | II | +++ |
| 07 | 51 | F | Breast | Lymph node metastatic and a little infiltrating ductal carcinoma | II | ++ |
| 08 | 39 | F | Breast | Lymph node metastatic infiltrating ductal carcinoma | II | ++ |
| 09 | 39 | F | Breast | Lymph node metastatic infiltrating ductal carcinoma | II | + |
| 10 | 52 | F | Breast | Lymph node metastatic infiltrating ductal carcinoma | II | + |
| 11 | 52 | F | Breast | Lymph node metastatic infiltrating ductal carcinoma | II | +++ |
| 12 | 58 | F | Breast | Lymph node metastatic infiltrating ductal carcinoma | II | +++ |
| 13 | 39 | F | Breast | Lymph node metastatic infiltrating ductal carcinoma | II | + |
| 14 | 49 | F | Breast | Lymph node metastatic infiltrating ductal carcinoma | II | ++ |
| 15 | 59 | F | Breast | Lymph node metastatic infiltrating ductal carcinoma | II | ++ |
| 16 | 46 | F | Breast | Infiltrating ductal carcinoma | I | +++ |
| 17 | 29 | F | Breast | Infiltrating ductal carcinoma | I | ++ |
| 18 | 55 | F | Breast | Infiltrating ductal carcinoma | II | +++ |
| 19 | 64 | F | Breast | Infiltrating ductal carcinoma | I | +++ |
| 20 | 30 | F | Breast | Infiltrating ductal carcinoma | II | ++ |
| 21 | 67 | F | Breast | Infiltrating ductal carcinoma | II | +++ |
| 22 | 52 | F | Breast | Infiltrating ductal carcinoma | II | ++ |
| 23 | 43 | F | Breast | Infiltrating ductal carcinoma | II | +++ |
| 24 | 53 | F | Breast | Infiltrating ductal carcinoma | II | + |
| 25 | 48 | F | Breast | Infiltrating ductal carcinoma | II | ++ |
| 26 | 53 | F | Breast | Infiltrating ductal carcinoma | II | +++ |
| 27 | 68 | F | Breast | Infiltrating ductal carcinoma | II | +++ |
| 28 | 38 | F | Breast | Infiltrating ductal carcinoma | II | +++ |
| 29 | 44 | F | Breast | Infiltrating ductal carcinoma | II | ++ |
| 30 | 38 | F | Breast | Infiltrating ductal carcinoma | II | ++ |
| 31 | 32 | F | Breast | Infiltrating ductal carcinoma | II | +++ |
| 32 | 52 | F | Breast | Infiltrating ductal carcinoma | II | ++ |
| 33 | 42 | F | Breast | Infiltrating ductal carcinoma | II | + |
| 34 | 44 | F | Breast | Infiltrating ductal carcinoma | II | +++ |
| 35 | 63 | F | Breast | Infiltrating ductal carcinoma | II | +++ |
| 36 | 45 | F | Breast | Infiltrating ductal carcinoma | II | + |
| 37 | 63 | F | Breast | Infiltrating ductal carcinoma | II | +++ |
| 38 | 54 | F | Breast | Infiltrating ductal carcinoma | II | +++ |
| 39 | 54 | F | Breast | Infiltrating ductal carcinoma | II | ++ |
| 40 | 43 | F | Breast | Infiltrating ductal carcinoma | II | ++ |
| 41 | 50 | F | Breast | Infiltrating ductal carcinoma | II | +++ |
| 42 | 45 | F | Breast | Infiltrating ductal carcinoma | II | +++ |
| 43 | 63 | F | Breast | Infiltrating ductal carcinoma | III | +++ |
| 44 | 65 | F | Breast | Infiltrating ductal carcinoma | II | ++ |
| 45 | 71 | F | Breast | Infiltrating ductal carcinoma | II | +++ |
| 46 | 52 | F | Breast | Infiltrating ductal carcinoma | II | ++ |
| 47 | 41 | F | Breast | Infiltrating ductal carcinoma | II | ++ |
| 48 | 45 | F | Breast | Infiltrating ductal carcinoma | II | +++ |
| 49 | 42 | F | Breast | Infiltrating ductal carcinoma | III | +++ |
| 50 | 44 | F | Breast | infiltrating lobular carcinoma | - | ++ |
| 51 | 47 | F | Breast | infiltrating lobular carcinoma | - | ++ |
| 52 | 51 | F | Breast | A little infiltrating lobular carcinoma | - | + |
| 53 | 59 | F | Breast | infiltrating lobular carcinoma | - | + |
| 54 | 38 | F | Breast | infiltrating lobular carcinoma | - | + |
| 55 | 47 | F | Breast | infiltrating lobular carcinoma | - | + |
| 56 | 19 | F | Breast | infiltrating lobular carcinoma | - | + |
| 57 | 48 | F | Breast | A little infiltrating lobular carcinoma | - | + |
| 58 | 45 | F | Breast | infiltrating lobular carcinoma | - | + |
| 59 | 38 | F | Breast | infiltrating lobular carcinoma | - | + |
| 60 | 36 | F | Breast | intraductal carcinoma | - | ++ |
| 61 | 68 | F | Breast | intraductal carcinoma | - | +++ |
| 62 | 54 | F | Breast | intraductal carcinoma | - | ++ |
| 63 | 45 | F | Breast | intraductal carcinoma | - | ++ |
| 64 | 46 | F | Breast | intraductal carcinoma | - | + |
| 65 | 52 | F | Breast | intraductal carcinoma | - | + |
| 66 | 47 | F | Breast | mammary adenosis | - | + |
| 67 | 48 | F | Breast | Breast tissue | - | + |
| 68 | 47 | F | Breast | intraductal carcinoma with early invasion | - | +++ |
| 69 | 49 | F | Breast | intraductal carcinoma | - | + |
| 70 | 45 | F | Breast | intraductal carcinoma | - | +++ |
| 71 | 64 | F | Breast | intraductal carcinoma | - | +++ |
| 72 | 55 | F | Breast | intraductal carcinoma | - | ++ |
| 73 | 48 | F | Breast | lobular carcinoma in situ | - | ++ |
| 74 | 44 | F | Breast | fibroadenoma | - | ++ |
| 75 | 23 | F | Breast | fibroadenoma | - | ++ |
| 76 | 45 | F | Breast | fibroadenoma | - | ++ |
| 77 | 19 | F | Breast | fibroadenoma | - | ++ |
| 78 | 23 | F | Breast | fibroadenoma | - | + |
| 79 | 34 | F | Breast | fibroadenoma | - | + |
| 80 | 25 | F | Breast | fibroadenoma | - | + |
| 81 | 48 | F | Breast | mammary adenosis with duct hyperplasia | - | + |
| 82 | 38 | F | Breast | mammary adenosis with duct hyperplasia | - | + |
| 83 | 41 | F | Breast | moderate hyperplasia of mammary duct | - | + |
| 84 | 40 | F | Breast | Atypical intraductal hyperplasia | II | +++ |
| 85 | 76 | F | Breast | mammary adenosis with duct hyperplasia | - | ++ |
| 86 | 37 | F | Breast | mammary adenosis with duct hyperplasia | - | ++ |
| 87 | 45 | F | Breast | mammary adenosis with moderate duct hyperplasia | - | ++ |
| 88 | 45 | F | Breast | mammary adenosis | - | + |
| 89 | 39 | F | Breast | mammary adenosis | - | + |
| 90 | 40 | F | Breast | mammary adenosis | - | + |
| 91 | 35 | F | Breast | mammary adenosis | - | + |
| 92 | 39 | F | Breast | chronic inflammation in mammary stroma with a little tumor tissue | - | + |
| 93 | 44 | F | Breast | chronic inflammation in mammary tissue | - | + |
| 94 | 43 | F | Breast | plasma cell mastitis | - | + |
| 95 | 43 | F | Breast | Breast tissue | - | + |
| 96 | 42 | F | Breast | Breast tissue | - | + |
| 97 | 40 | F | Breast | Breast tissue | - | + |
| 98 | 44 | F | Breast | Breast tissue | - | + |
| 99 | 45 | F | Breast | Breast tissue | - | + |

Note: “-” in Grade means no grading available.
