# Supplementary material for: Oncoprotein HBXIP enhances HOXB13 acetylation and co-activates HOXB13 to confer tamoxifen resistance in breast cancer
Source: J Hematol Oncol. 2018 Feb 23;11:26. doi: 10.1186/s13045-018-0577-5 (PMC5824486; doi:10.1186/s13045-018-0577-5)
Supplement: Supplementary file 3 — Table S3. Clinical characteristics of 34 ER+ breast cancer tissue samples. (DOCX 19 kb) [file 13045_2018_577_MOESM3_ESM.docx]

**Table S4**. Clinical characteristics of 34 ER+ breast cancer tissue samples

| **No.** | **Age** | **Sex** | **Organ** | **Pathology diagnosis** | **Grade** | **ER** |
| --- | --- | --- | --- | --- | --- | --- |
| 01 | 41 | F | Breast | Nonspecific infiltrating ductal carcinoma | I | + |
| 02 | 47 | F | Breast | A little nonspecific infiltrating ductal carcinoma | I | + |
| 03 | 57 | F | Breast | A little nonspecific infiltrating ductal carcinoma | II | ++ |
| 04 | 66 | F | Breast | Nonspecific infiltrating ductal carcinoma | I | + |
| 05 | 40 | F | Breast | A little nonspecific infiltrating ductal carcinoma | I | + |
| 06 | 52 | F | Breast | A little nonspecific infiltrating ductal carcinoma | I | ++ |
| 07 | 44 | F | Breast | Nonspecific infiltrating ductal carcinoma | II | + |
| 08 | 65 | F | Breast | A little nonspecific infiltrating ductal carcinoma | II | +++ |
| 09 | 56 | F | Breast | Nonspecific infiltrating ductal carcinoma | I-II | ++ |
| 10 | 55 | F | Breast | Nonspecific infiltrating ductal carcinoma | II | ++ |
| 11 | 57 | F | Breast | Nonspecific infiltrating ductal carcinoma | II | + |
| 12 | 76 | F | Breast | Nonspecific infiltrating ductal carcinoma | I | ++ |
| 13 | 58 | F | Breast | Nonspecific infiltrating ductal carcinoma | II | + |
| 14 | 47 | F | Breast | Nonspecific infiltrating ductal carcinoma | I | + |
| 15 | 57 | F | Breast | A little nonspecific infiltrating ductal carcinoma | II | +++ |
| 16 | 50 | F | Breast | Nonspecific infiltrating ductal carcinoma | II | +++ |
| 17 | 49 | F | Breast | Nonspecific infiltrating ductal carcinoma | II | + |
| 18 | 35 | F | Breast | A little nonspecific infiltrating ductal carcinoma | II | ++ |
| 19 | 47 | F | Breast | Nonspecific infiltrating ductal carcinoma | I | + |
| 20 | 59 | F | Breast | A little nonspecific infiltrating ductal carcinoma | III | ++ |
| 21 | 40 | F | Breast | Nonspecific infiltrating ductal carcinoma | II | + |
| 22 | 60 | F | Breast | Nonspecific infiltrating ductal carcinoma | I | + |
| 23 | 26 | F | Breast | Nonspecific infiltrating ductal carcinoma | I | ++ |
| 24 | 49 | F | Breast | Nonspecific infiltrating ductal carcinoma | I-II | ++ |
| 25 | 46 | F | Breast | Infiltrating ductal carcinoma | III | +++ |
| 26 | 45 | F | Breast | Nonspecific infiltrating ductal carcinoma | II | + |
| 27 | 46 | F | Breast | Infiltrating ductal carcinoma | II | +++ |
| 28 | 59 | F | Breast | Infiltrating ductal carcinoma | III | +++ |
| 29 | 64 | F | Breast | Infiltrating ductal carcinoma | II | ++ |
| 30 | 38 | F | Breast | Infiltrating ductal carcinoma | II | +++ |
| 31 | 37 | F | Breast | Infiltrating ductal carcinoma | III | ++ |
| 32 | 53 | F | Breast | Nonspecific infiltrating ductal carcinoma | II | ++ |
| 33 | 54 | F | Breast | Infiltrating ductal carcinoma | II | +++ |
| 34 | 49 | F | Breast | Infiltrating ductal carcinoma | II | +++ |

Note: “-” in Grade means no grading available.
