# Supplementary material for: Oncoprotein HBXIP enhances HOXB13 acetylation and co-activates HOXB13 to confer tamoxifen resistance in breast cancer
Source: J Hematol Oncol. 2018 Feb 23;11:26. doi: 10.1186/s13045-018-0577-5 (PMC5824486; doi:10.1186/s13045-018-0577-5)

Liu BW, et al., Figure S1

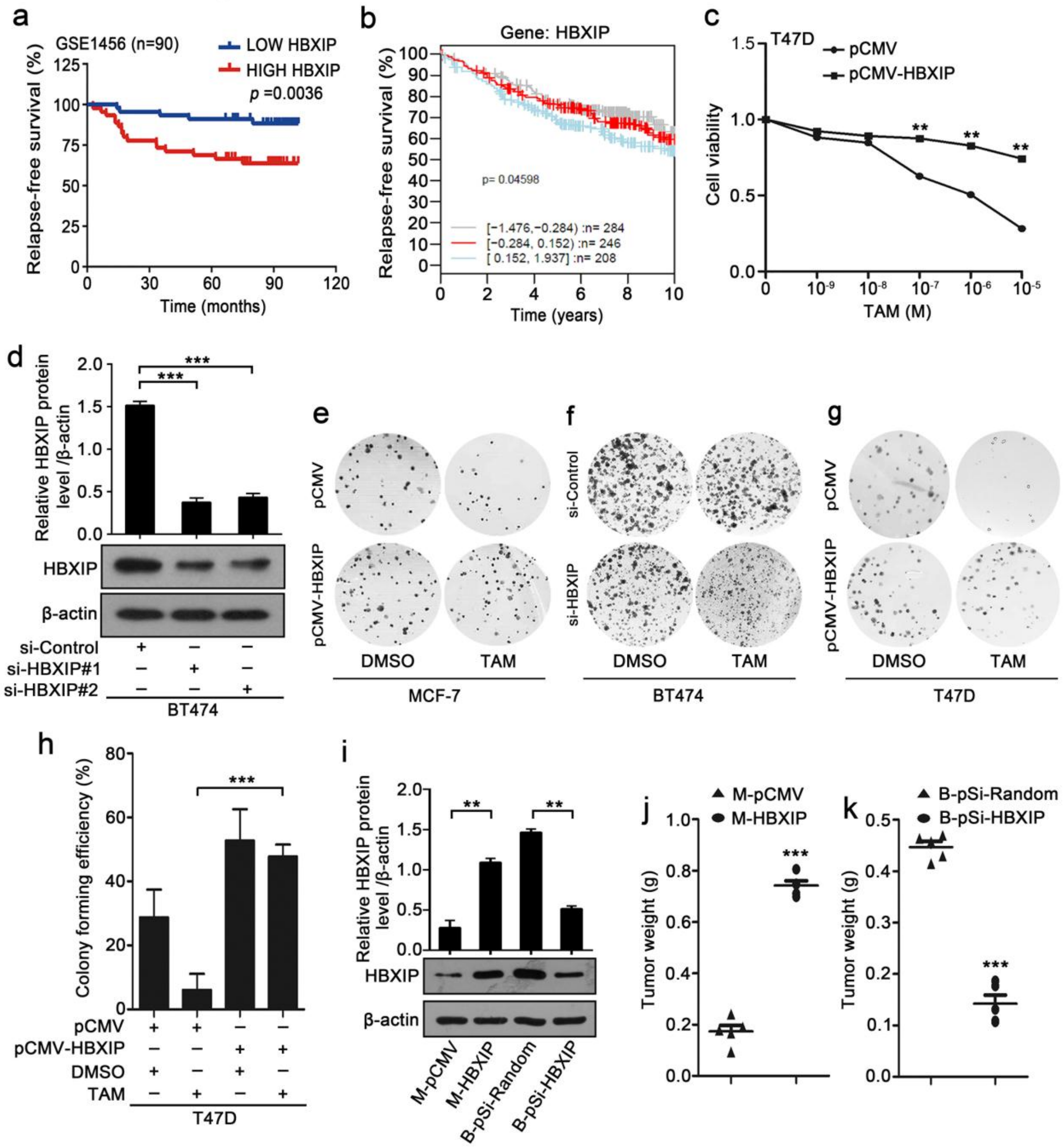

Liu BW, et al., Figure S2

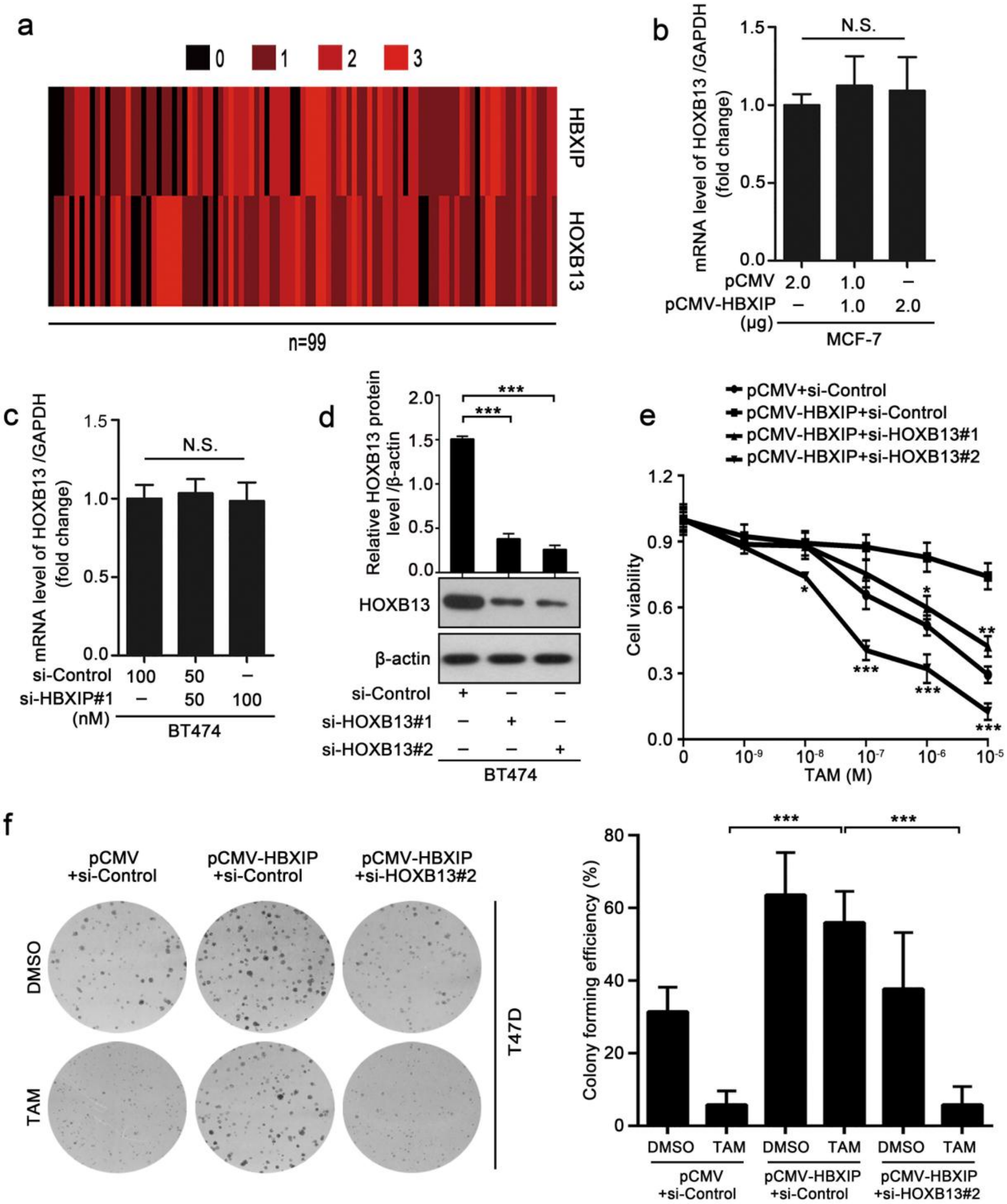

Liu BW, et al., Figure S3

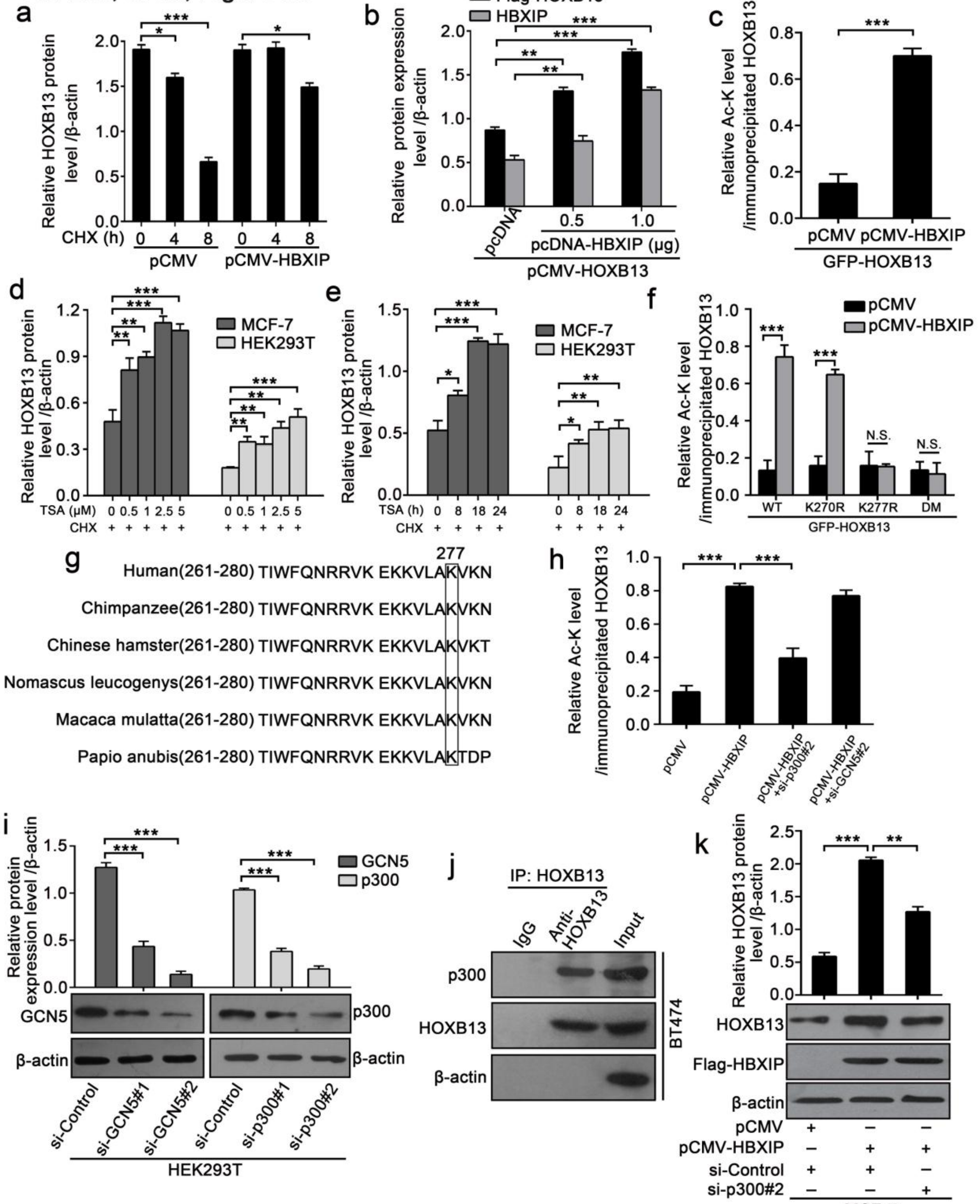

Liu BW, et al., Figure S4

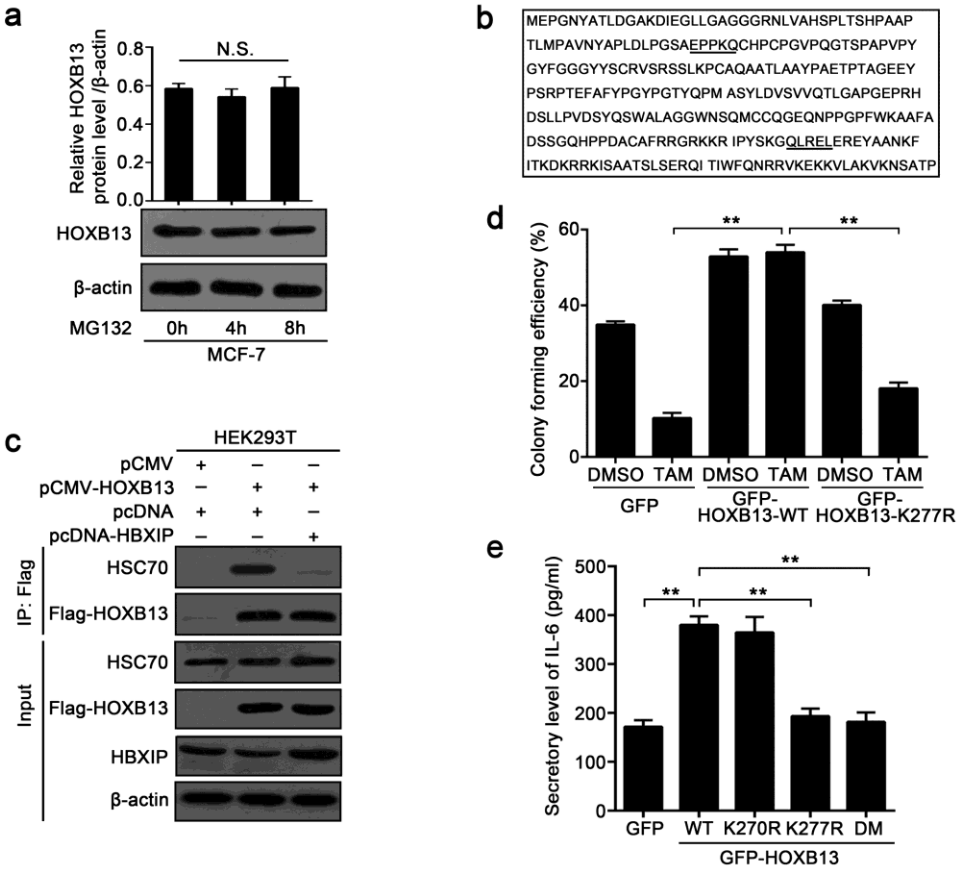

Liu BW, et al., Figure S5

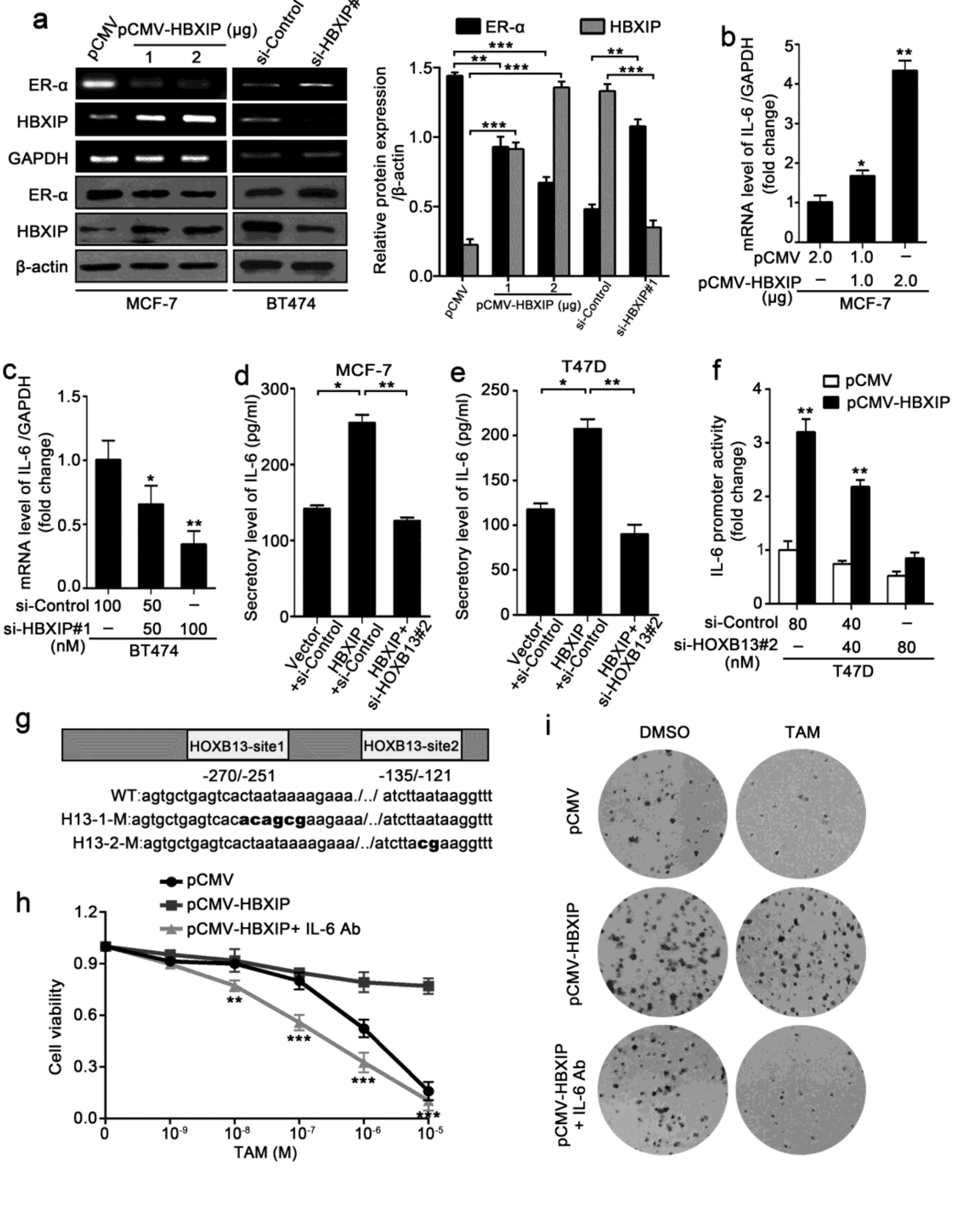

Liu BW, et al.,Figure S6

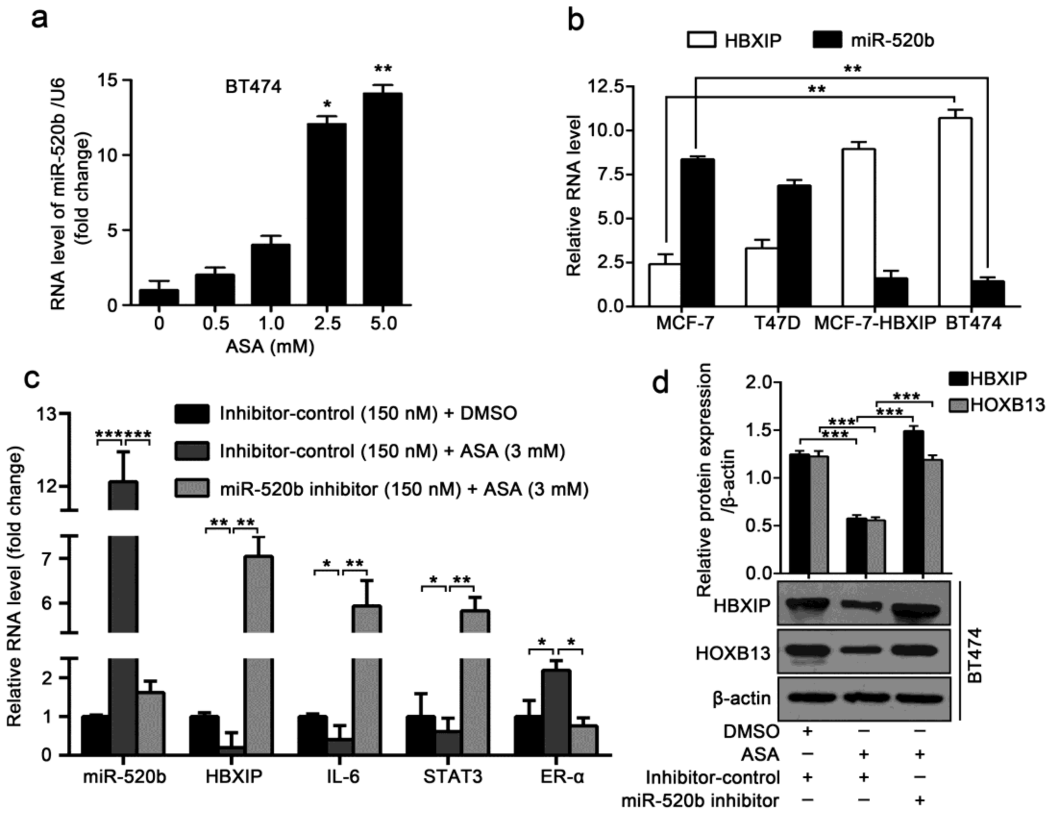

Liu BW, et al.,Figure S7

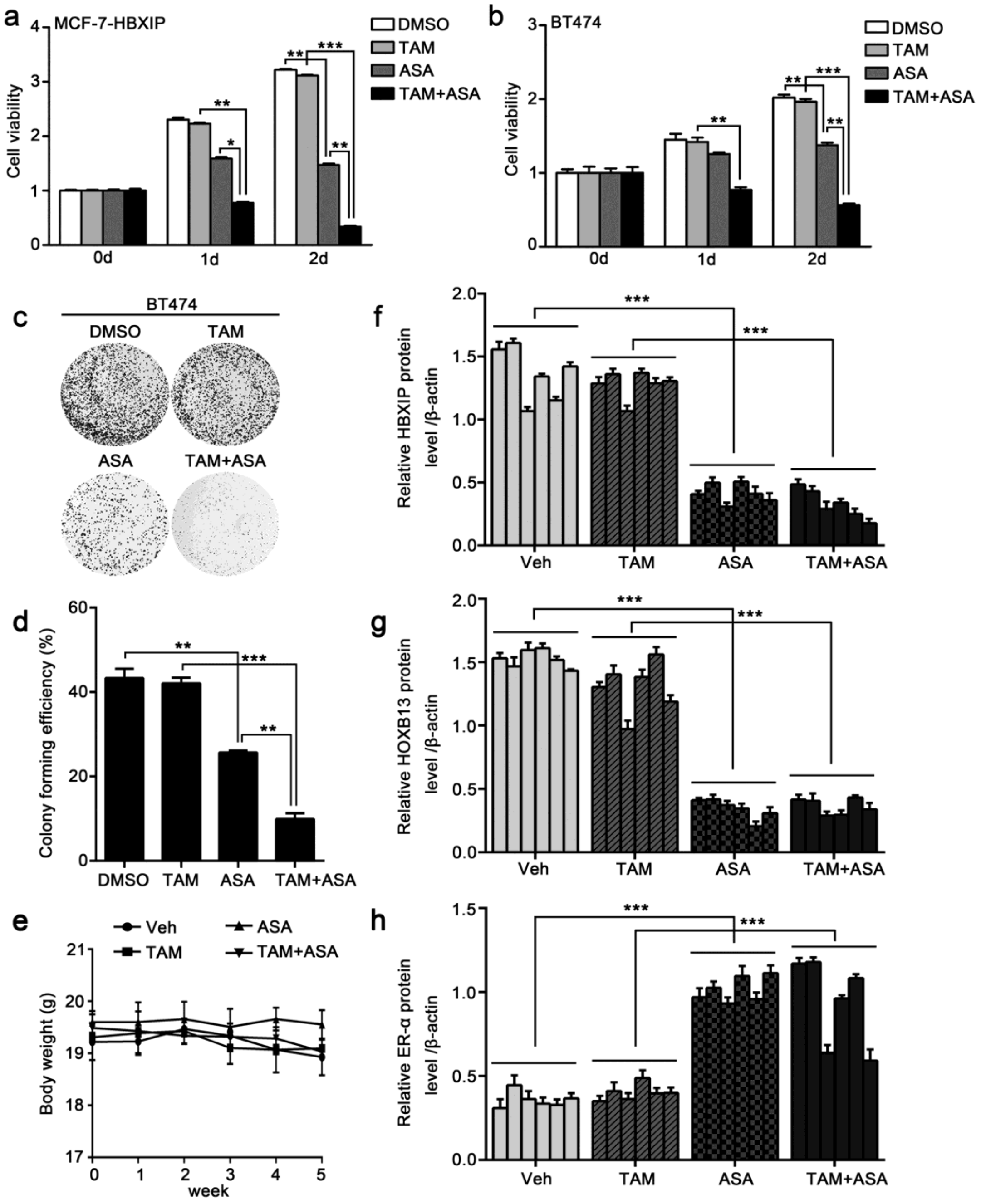

Liu BW, et al., Figure S8

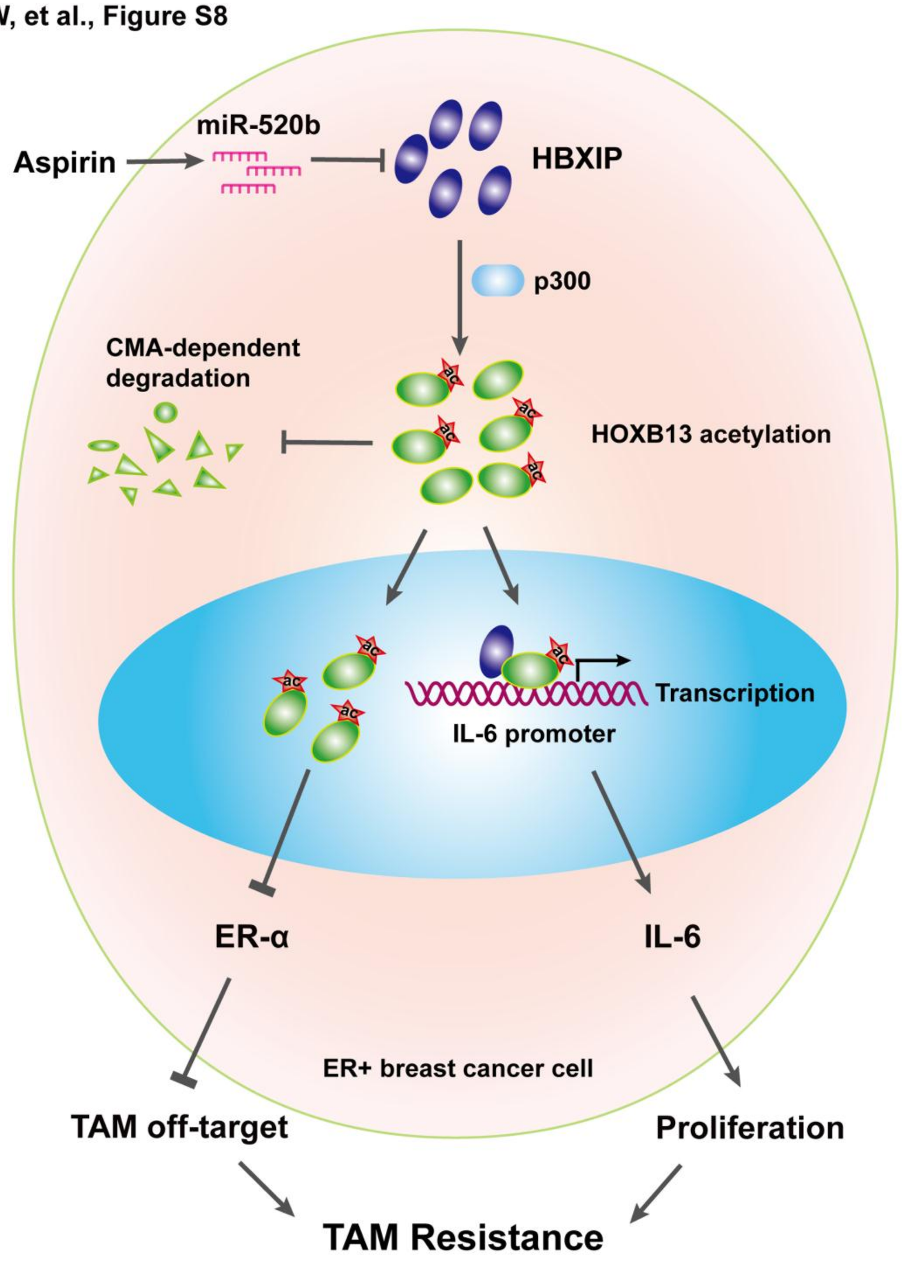

Supplement: Supplementary file 4 — Figure S1. HBXIP contributes to TAM resistance in breast cancer. Figure S2. HBXIP induces TAM resistance by increasing the protein level of HOXB13. Figure S3. HBXIP enhances acetylation of HOXB13 at K277 site via acetylase p300. Figure S4. HBXIP-enhanced acetylation of HOXB13 stabilizes HOXB13 in the facilitation of TAM resistance. Figure S5. HBXIP co-activates HOXB13 to stimulate IL-6 transcription. Figure S6. ASA suppresses HBXIP/HOXB13 axis by reducing HBXIP expression. Figure S7. ASA-inhibited HBXIP/HOXB13 axis contributes to the reversal of TAM resistance. Figure S8. Diagram of working model. (ZIP 1058 kb) [file 13045_2018_577_MOESM4_ESM.zip › Additional File 1.pdf]
