# Supplementary material for: Oncoprotein HBXIP enhances HOXB13 acetylation and co-activates HOXB13 to confer tamoxifen resistance in breast cancer
Source: J Hematol Oncol. 2018 Feb 23;11:26. doi: 10.1186/s13045-018-0577-5 (PMC5824486; doi:10.1186/s13045-018-0577-5)
Supplement: Supplementary file 4 — Figure S1. HBXIP contributes to TAM resistance in breast cancer. Figure S2. HBXIP induces TAM resistance by increasing the protein level of HOXB13. Figure S3. HBXIP enhances acetylation of HOXB13 at K277 site via acetylase p300. Figure S4. HBXIP-enhanced acetylation of HOXB13 stabilizes HOXB13 in the facilitation of TAM resistance. Figure S5. HBXIP co-activates HOXB13 to stimulate IL-6 transcription. Figure S6. ASA suppresses HBXIP/HOXB13 axis by reducing HBXIP expression. Figure S7. ASA-inhibited HBXIP/HOXB13 axis contributes to the reversal of TAM resistance. Figure S8. Diagram of working model. (ZIP 1058 kb) [file 13045_2018_577_MOESM4_ESM.zip › Additional file 2 Additional figure legends .docx]

**Additional Figure Legends**

**Figure S1** HBXIP contributes to TAM resistance in breast cancer. (**a**) Relapse-free survival analysis of 90 mono-TAM treated breast cancer patients with low versus high HBXIP expression (Gehan-Breslow-Wilcoxon test, *p*=0.0036). (**b**) Relapse-free survival analysis of 1225 ER+ breast cancer patients by HBXIP expression (low vs. moderate vs. high, *p* =

0.04598). (**c**) Cell viability assay with T47D cells treated with corresponding doses of TAM after being transiently transfected with the indicated plasmids. (**d**) Interference efficiencies of si-HBXIP#1 and si-HBXIP#2 examined by immunoblotting analysis in BT474 cells (lower panel). The upper panel is the quantification of the intensity relative to β-actin. (**e and f**) Colony photograph of MCF-7 cells (**e**) and BT474 cells (**f**) treated with DMSO or TAM (1 μM) after being transiently transfected with the indicated plasmids or siRNA. (**g and h**) Colony photograph (**g**) and colony forming efficiency (**h**) of T47D cells treated with DMSO or TAM (1 μM) after being transiently transfected with the indicated plasmids. (**i**) Protein level of HBXIP in the indicated cell lines examined by immunoblotting analysis (lower panel) and the quantification of the intensity relative to β-actin (upper panel). (**j and k**) Weights of the xenograft tumors derived from M-pCMV or M-HBXIP cells (**j**), B-pSi-Random or B-pSi-HBXIP cells (**k**). Each group, n=5. All experiments were repeated at least three times. Error bars represent ±SD. **P* < 0.05, ***P* < 0.01, ****P* < 0.001 by 2-tailed Student’s *t-*test.

**Figure S2** HBXIP induces TAM resistance by increasing the protein level of HOXB13**.** (**a**) Heatmap of the expression level of HBXIP and HOXB13 in ER+ breast tissue microarray. Numbers 0, 1, 2 or 3 represents the negative, weak, moderate or intense staining, respectively. **(b and c)** qRT–PCR assay of HOXB13 expression in MCF-7 cells **(b)**, BT474 cells **(c)** being transiently transfected with indicated plasmids or siRNA. N.S., not significant. (**d**) Interference efficiencies of si-HOXB13#1 and si-HOXB13#2 examined by immunoblotting analysis in BT474 cells (lower panel). The upper panel is the quantification of the intensity relative to β-actin. (**e**) Cell viability assay with T47D cells treated with the indicated concentrations of TAM after being transiently transfected with the displayed plasmids or siRNA. Error bars represent ±SD. **P* < 0.05, ***P* < 0.01, ****P* < 0.001 (HBXIP compared with HBXIP+si-HOXB13) by 2-tailed Student’s *t-*test. (**f**) Colony photograph and colony forming efficiency of T47D cells treated with DMSO or TAM (1μM) after being transiently transfected with the displayed plasmids or siRNA. All experiments were repeated at least three times. Error bars represent ±SD. **P* < 0.05, ***P* < 0.01, ****P* < 0.001 by 2-tailed Student’s *t-*test.

**Figure S3** HBXIP enhances acetylation of HOXB13 at K277 site *via* acetylase p300. (**a**) The quantification of the intensity relative to β-actin in Figure 3a. (**b**) The quantification of the intensity relative to β-actin in Figure 3b. (**c**) The quantification of the intensity relative to immunoprecipitated HOXB13 in Figure 3c. (**d** and **e**) The quantifications of the intensity relative to β-actin in Figure 3d (**d**), and 3e (**e**). (**f**) The quantification of the intensity relative to immunoprecipitated HOXB13 in Figure 3f. (**g**) Sequence alignment of the putative acetylation site K277 in HOXB13 from different species. (**h**) The quantification of the intensity relative to immunoprecipitated HOXB13 in Figure 3g. (**i**) Interference efficiencies of si-p300#1, si-p300#2, si-GCN5#1 and si-GCN5#2 examined by immunoblotting analysis in HEK293T cells (lower panel). The upper panel is the quantification of the intensity relative to β-actin. (**j**) Interaction of endogenous HOXB13 with p300 examined by Co-IP assay in BT474 cells. (**k**) Immunoblotting analysis of HOXB13 in MCF-7 cells being transiently transfected with indicated plasmids or siRNA (lower panel). The protein level of HBXIP was examined by the anti-Flag antibody. The upper panel is the quantification of the intensity relative to β-actin. All experiments were repeated at least three times. Error bars represent ±SD. **P* < 0.05, ***P* < 0.01, ****P* < 0.001 by 2-tailed Student’s *t-*test.

**Figure S4** HBXIP-enhanced acetylation of HOXB13 stabilizes HOXB13 in facilitation of TAM resistance. (**a**) Immunoblotting analysis of HOXB13 in MCF-7 cells time-dependently treated with 50 μM MG132 (lower panel). The upper panel is the quantification of the intensity relative to β-actin. (**b**) The lysosomal targeting motifs in HOXB13 peptide sequence, which were highlighted with lineation. (**c**) Interaction of Flag-HOXB13 with HSC70 was analyzed by Co-IP assay in HEK293T cells. The cells were transiently transfected with pCMV or pCMV-HOXB13 accompanied with pcDNA or pcDNA-HBXIP. (**d**) Colony forming efficiency of MCF-7 cells treated with DMSO or TAM (1μM) after being transiently transfected with displayed plasmids. (**e**) ELISA showing the secretion of IL-6 in MCF-7 cells after being transiently transfected with the GFP empty vector, WT or different mutants based on GFP-HOXB13, respectively. All experiments were repeated at least three times. Error bars represent ±SD. ***P* < 0.01, ****P* < 0.001 by 2-tailed Student’s *t-*test.

**Figure S5** HBXIP co-activates HOXB13 to stimulate IL-6 transcription. (**a**) The mRNA and protein levels of ER-α were separately detected by RT-PCR and immunoblotting analysis in MCF-7 cells and BT474 cells (left panel). Both cells were transiently transfected with the corresponding plasmids or siRNA. The right panel is the quantification of the intensity relative to β-actin in immunoblotting analysis. (**b and c**) qRT-PCR assay of IL-6 expression in MCF-7 cells (**b**) and BT474 cells (**c**) which were transiently transfected with the indicated plasmids or siRNA. (**d and e**) ELISA showing the secretion of IL-6 in MCF-7 cells (**d**) and T47D cells (**e**) after being transiently transfected with pCMV (vector) or pCMV-HBXIP (HBXIP) companied with si-Control or si-HOXB13#2. (**f**) Luciferase reporter gene assay of IL-6 promoter activity in T47D cells after being transiently transfected with pCMV or pCMV-HBXIP accompanied with the indicated si-control or si-HOXB13#2. (**g**) The nucleotide mutations diagram of binding sites of HOXB13-site1 (H13-1-M) and HOXB13-site2 (H13-2-M). (**h**) Cell viability assay with MCF-7 cells treated with the indicated concentrations of TAM after being transiently transfected with the displayed plasmids with or without IL-6 neutralizing antibody (IL-6 Ab, 1:400 dilution). Error bars represent ±SD. ***P* < 0.01, ****P* < 0.001 (HBXIP compared with HBXIP + IL-6 Ab) by 2-tailed Student’s *t-*test. (**i**) Colony photograph of MCF-7 cells treated with DMSO or TAM (1μM) after being transiently transfected with the displayed plasmids with or without IL-6 neutralizing antibody (IL-6 Ab). All experiments were repeated at least three times. Error bars represent ±SD. **P* < 0.05, ***P* < 0.01, ****P* < 0.001 by 2-tailed Student’s *t-*test.

**Figure S6** ASA suppresses HBXIP/HOXB13 axis by reducing HBXIP expression. (**a**) qRT-RCR assay of miR-520b expression in BT474 cells treated with the indicated doses of ASA for 24 h. (**b**) qRT-RCR assay of HBXIP and miR-520b in the indicated breast cancer cell lines. The expression values were normalized against GAPDH or U6, respectively. (**c**) qRT-PCR assay of miR-520b, HBXIP, IL-6, STAT3 and ER-α in BT474 cells treated with DMSO or ASA for 24h after being transiently transfected with the inhibitor-control or miR-520b inhibitor for 24h. The expression values were normalized against GAPDH or U6, respectively. (**d**) Immunoblotting analysis of HBXIP and HOXB13 in BT474 cells treated with DMSO or ASA for 24h after being transiently transfected with the inhibitor-control or miR-520b inhibitor for 24h (lower panel). The upper panel is the quantification of the intensity relative to β-actin. All experiments were repeated at least three times. Error bars represent ±SD. **P* < 0.05, ***P* < 0.01, ****P* < 0.001 by 2-tailed Student’s *t-*test.

**Figure S7** ASA-inhibited HBXIP/HOXB13 axis contributes to the reversal of TAM resistance. (**a and b**) Cell viability assay with MCF-7-HBXIP (**a**) and BT474 cells (**b**) separately treated with DMSO, TAM (3 μM), ASA (2.5 mM) or combination of TAM and ASA (TAM+ASA) at the indicated time points. (**c and d**) Colony photograph **(c)** and colony forming efficiency **(d)** of BT474 cells separately treated with DMSO, TAM (3 μM), ASA (2.5 mM) or combination of TAM and ASA (TAM+ASA). (**e**) Body weight measurement for xenograft experiment shown in Figure 7a. (**f-h**) The quantification of the intensity relative to β-actin in Figure 7f. All experiments were repeated at least three times. Error bars represent ±SD. **P* < 0.05, ***P* < 0.01, ****P* < 0.001 by 2-tailed Student’s *t-*test.

**Figure S8** Diagram of working model. HBXIP induces TAM resistance in ER+ breast cancer through modulating HOXB13. HBXIP can prevent CMA-dependent degradation of HOXB13 through elevating its acetylation at K277 by acetylase p300, resulting in HOXB13 accumulation. Then, HBXIP inhibits the expression of ER-α by HOXB13, leading to TAM off-target, and acts as a co-activator of HOXB13 to stimulate IL-6 transcription, resulting in the acceleration of proliferation. ASA-mediated upregulation of miR-520b suppresses HBXIP expression, blocking the HBXIP/HOXB13 axis and restoring the sensitivity to TAM.
