# Supplementary material for: Oncoprotein HBXIP enhances HOXB13 acetylation and co-activates HOXB13 to confer tamoxifen resistance in breast cancer
Source: J Hematol Oncol. 2018 Feb 23;11:26. doi: 10.1186/s13045-018-0577-5 (PMC5824486; doi:10.1186/s13045-018-0577-5)
Supplement: Supplementary file 5 — Additional methods. (DOCX 27 kb) [file 13045_2018_577_MOESM5_ESM.docx]

**Additional methods**

**Chromatin immunoprecipitation**

According to the manufacturer’s instructions, Chromatin immunoprecipitation (ChIP) assay was performed using the EpiQuik^TM^ chromatinimmunoprecipitation kit from Epigentek Group Inc (Brooklyn, NY). The protein-DNA complexes were immunoprecipitated with anti-HBXIP, anti-HOXB13 antibody, or rabbit IgG (a negative control antibody). DNA from these samples was amplified by PCR and qPCR analysis. The primers were listed in Additional file 3: Table S1.

**Immunoblotting analysis**

Immunoblotting analysis was performed with the standard protocol. Briefly, cells were washed with cold PBS three times and lysed in RIPA cell lysis buffer (10 mmol/liter HEPES, pH 7.4, 0.15 mol/liter NaCl, 1 mmol/liter MgCl_2_, 1 mmol/liter CaCl_2_, 1 mmol/liter dithiothreitol, 0.1% SDS, 0.1% Nonidet P-40, and 20 μg/ml leupeptin). Equal amounts of total protein were loaded for immunoblotting. Following SDS-PAGE, resolved proteins were electrotransferred onto PVDF membranes (Millipore, USA). The membranes were blocked overnight in TBS containing 0.1% Tween 20 (TBST) and 7% skim milk. Membranes were then probed with primary antibody in TBST for 2 h at room temperature or overnight at 4 °C, followed by three 15-min TBST washes at room temperature. Blots were incubated with HRP-conjugated secondary antibody for 1 h and washed three times for 10 min with TBST prior to chemiluminescence detection using ECL substrate (Millipore). The Image J software was used to quantify the intensity in immunoblotting analysis. All experiments were repeated three times.

**Luciferase reporter gene assay**

Cells (2 × 10^4^ cells per well) grown in 24-well plates were co-transfected with IL-6 luciferase reporter plasmid (0.2 μg) and pRL-TK normalization construct (0.1 μg) using Lipofectamine 2000 (Invitrogen). The pCMV-HBXIP plasmid (0.1–0.2 μg) was co-transfected with reporter plasmids to overexpress HBXIP. siRNAs targeting HBXIP or HOXB13 (40–100 nM) and the reporter plasmids were co-transfected into the cells. Cells were harvested after transfection for 24h and luciferase reporter gene assay was implemented using the Dual-Luciferase Reporter Assay System (Promega) according to the manufacturer’s instructions [[1](#_ENREF_1)].

**Reverse-transcription polymerase chain reaction (RT-PCR) and quantitative reverse-transcription polymerase chain reaction (qRT-PCR)**

RT-PCR and qRT-PCR assays were carried out as previously described [[2](#_ENREF_2)]. All primers were listed in Additional file 3: Table S1.

**ELISA**

IL-6 level was determined using a commercially available assay (human IL-6 ELISA kit, Neobiocience, China) according to the manufacturer’s instructions. Breast cancer cells were seeded in 6-well plates and transfected or treated with indicated plasmid or reagent. When the cultures reached 70–80% confluence, fresh medium was applied and collected after 24 h of incubation. IL-6 levels were determined by ELISA reader system (Labsystem, Multi-skan Ascent). Triplicate cultures of cells were tested for each experimental condition.

**Cell viability assay**

Cell viability assay was carried out using 3-(4, 5-dimethylthiazol-2-yl)-2, 5 diphenyltetrazolium bromide (MTT) reagent (Sigma) as described previously [[1](#_ENREF_1)]. In brief, transfected cells were trypsinized, counted, and plated into duplicate wells of 96-well plates at a density of 10^3^ cells per well. Each group was carried out with six wells and repeated three times. After incubation for indicated reagents, MTT was added directly to each well. Incubation was continued for 4h, and then the supernatant was removed and dimethyl sulfoxide (DMSO) was added to stop the reaction. Absorbance (492-620 nm) was measured using a reader system (Labsystem, Multiskan Ascent). Reagents used as follows: 4OH-TAM (Sigma-Aldrich), ASA (Sigma-Aldrich).

**Colony formation assay**

The transfected cells were trypsinized and cultured in 6-well plate. The medium was respectively added with corresponding reagents. The medium was replaced every three days. After growth for 10 days, cells were washed with PBS for at least 2 times and fixed in methanol for 20 min at room temperature; finally cells were stained with crystal violet. The number of colonies was counted and the colony forming efficiency was determined with the formula: colony forming efficiency = number of colonies counted/number of cells plated × 100%.

**Immunofluorescence staining**

Cells were grown on acid-treated glass coverslips as described elsewhere. Treated cells were fixed with ice-cold 4% paraformaldehyde for 10 min, washed three times with phosphate-buffered saline (PBS) and permeabilized with 0.1% Triton X-100 in PBS for 20 min. After washing three times with 0.05% Tween-20 in PBS, samples were blocked in PBS containing 3% BSA for 1 h. Cells were incubated with primary antibodies such as rabbit anti-HBXIP (Abcam, UK), rabbit anti-HOXB13 (Abcam), and then the secondary antibodies such as Alexa Fluor 488 goat anti-rabbit IgG (Invitrogen, USA), Alexa Fluor 594 goat anti-rabbit IgG (Invitrogen); and DAPI staining. For observation of localization of HBXIP and HOXB13, the cells were visualized using laser scanning confocal microscopy (Leica,Germany).

**References**

1. Shan C, Xu F, Zhang S, You J, You X, Qiu L, Zheng J, Ye L, Zhang X: Hepatitis B virus X protein promotes liver cell proliferation via a positive cascade loop involving arachidonic acid metabolism and p-ERK1/2. *Cell Res* 2010, 20:563-575.

2. Liu S, Li L, Zhang Y, Zhang Y, Zhao Y, You X, Lin Z, Zhang X, Ye L: The oncoprotein HBXIP uses two pathways to up-regulate S100A4 in promotion of growth and migration of breast cancer cells. *J Biol Chem* 2012, 287:30228-30239.
